# Supplementary material for: Ecological suitability of Japanese encephalitis virus in Australia: A modelling analysis of vector-host transmission dynamics to potential spillover in humans
Source: PLoS Negl Trop Dis. 2025 Nov 17;19(11):e0013722. doi: 10.1371/journal.pntd.0013722 (PMC12654935; doi:10.1371/journal.pntd.0013722)
Supplement: S2 Table — (DOCX) [file pntd.0013722.s002.docx]

**S2 Table: Parameter assumptions used in the modelling based on published studies [1, 2]**

| **Species** | **Parameter** | **Low** | **Average** | **High** |
| --- | --- | --- | --- | --- |
| *Culex annulirostris* | Bloodfeeding (Ardeidae) | 0.0557 | 0.1563 | 0.2686 |
|  | Bloodfeeding (Pigs) | 0.0174 | 0.0749 | 0.3457 |
|  | Bloodfeeding (Feral Pigs) | 0.0174 | 0.0749 | 0.3457 |
|  | Infection Probability | 0.8 | 0.91 | 1 |
|  | Transmission Potential | 0.12 | 0.5133 | 0.81 |
| *Culex quinquefasciatus* | Bloodfeeding (Ardeidae) | 0.319 | 0.4311 | 0.7855 |
|  | Bloodfeeding (Pigs) | 0.0018 | 0.0049 | 0.0128 |
|  | Bloodfeeding (Feral Pigs) | 0.0018 | 0.0049 | 0.0128 |
|  | Infection Probability | 0.56 | 0.77 | 0.98 |
|  | Transmission Potential | 0 | 0.25 | 0.5 |

[1] Stephenson EB, Murphy AK, Jansen CC, Peel AJ, McCallum H. Interpreting mosquito feeding patterns in Australia through an ecological lens: an analysis of blood meal studies. Parasites & vectors. 2019;12:1-11.

[2] van den Hurk AF, Skinner E, Ritchie SA, Mackenzie JS. The emergence of Japanese encephalitis virus in Australia in 2022: existing knowledge of mosquito vectors. Viruses. 2022;14(6):1208.
