# Supplementary figures and images for: Ecological suitability of Japanese encephalitis virus in Australia: A modelling analysis of vector-host transmission dynamics to potential spillover in humans
Source: PLoS Negl Trop Dis. 2025 Nov 17;19(11):e0013722. doi: 10.1371/journal.pntd.0013722 (PMC12654935; doi:10.1371/journal.pntd.0013722)

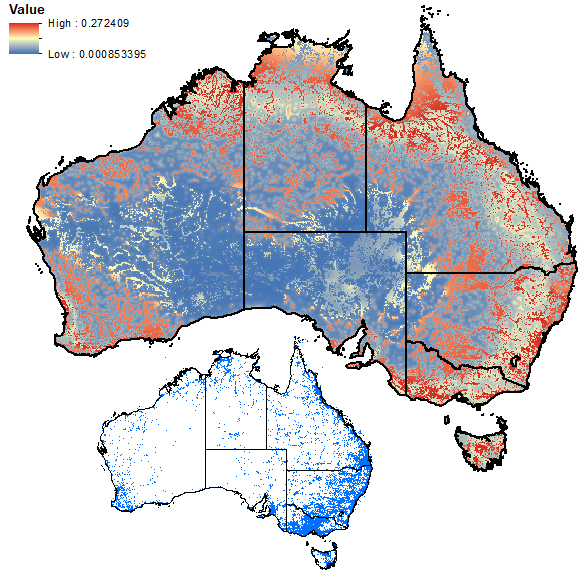

Supplement: S1 Fig — Small map panel displays observed occurrence data from 2013–2023 (Atlas of Living Australia) used in the maximum entropy model component. (TIF) [file pntd.0013722.s003.tif]

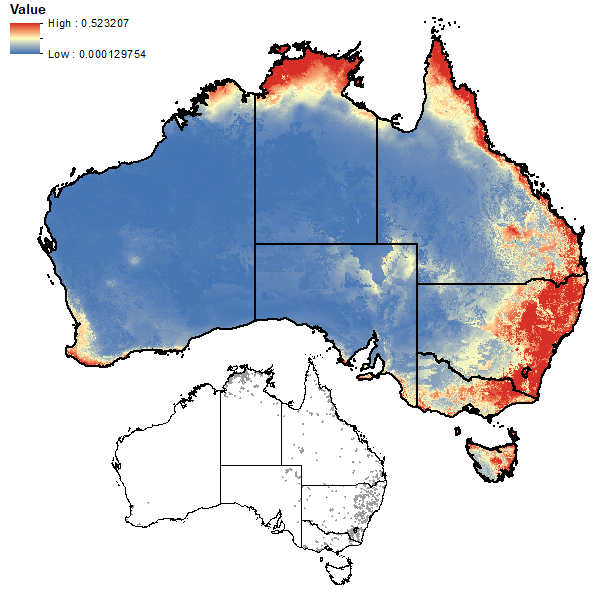

Supplement: S2 Fig — Small panel displays observed occurrence from 2018–2023 (Atlas of Living Australia) used in the maximum entropy model. (TIF) [file pntd.0013722.s004.tif]

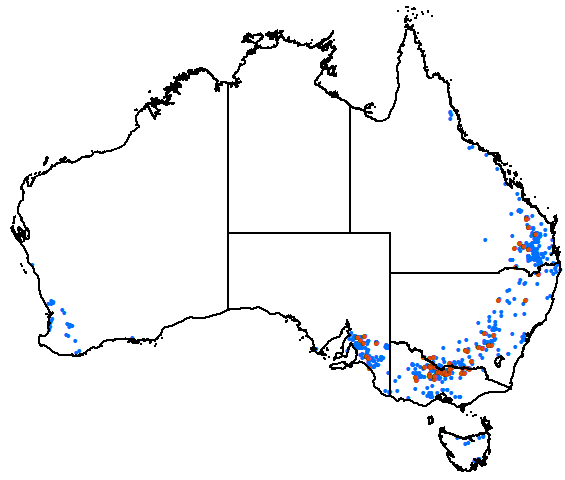

Supplement: S3 Fig — (TIF) [file pntd.0013722.s005.tif]

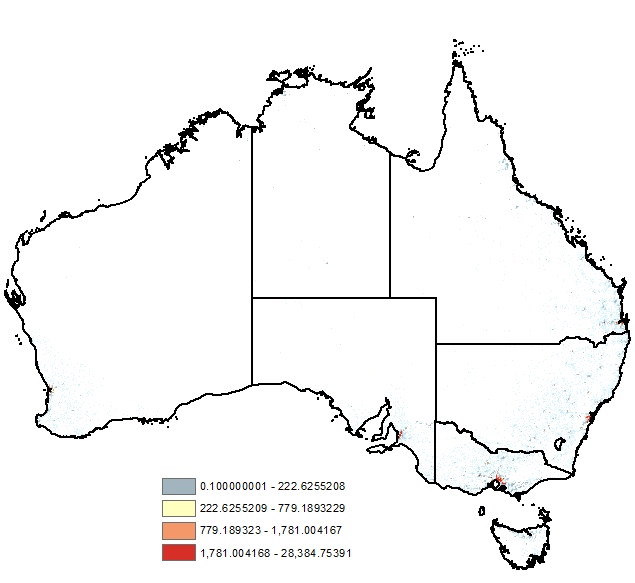

Supplement: S4 Fig — (TIF) [file pntd.0013722.s006.tif]

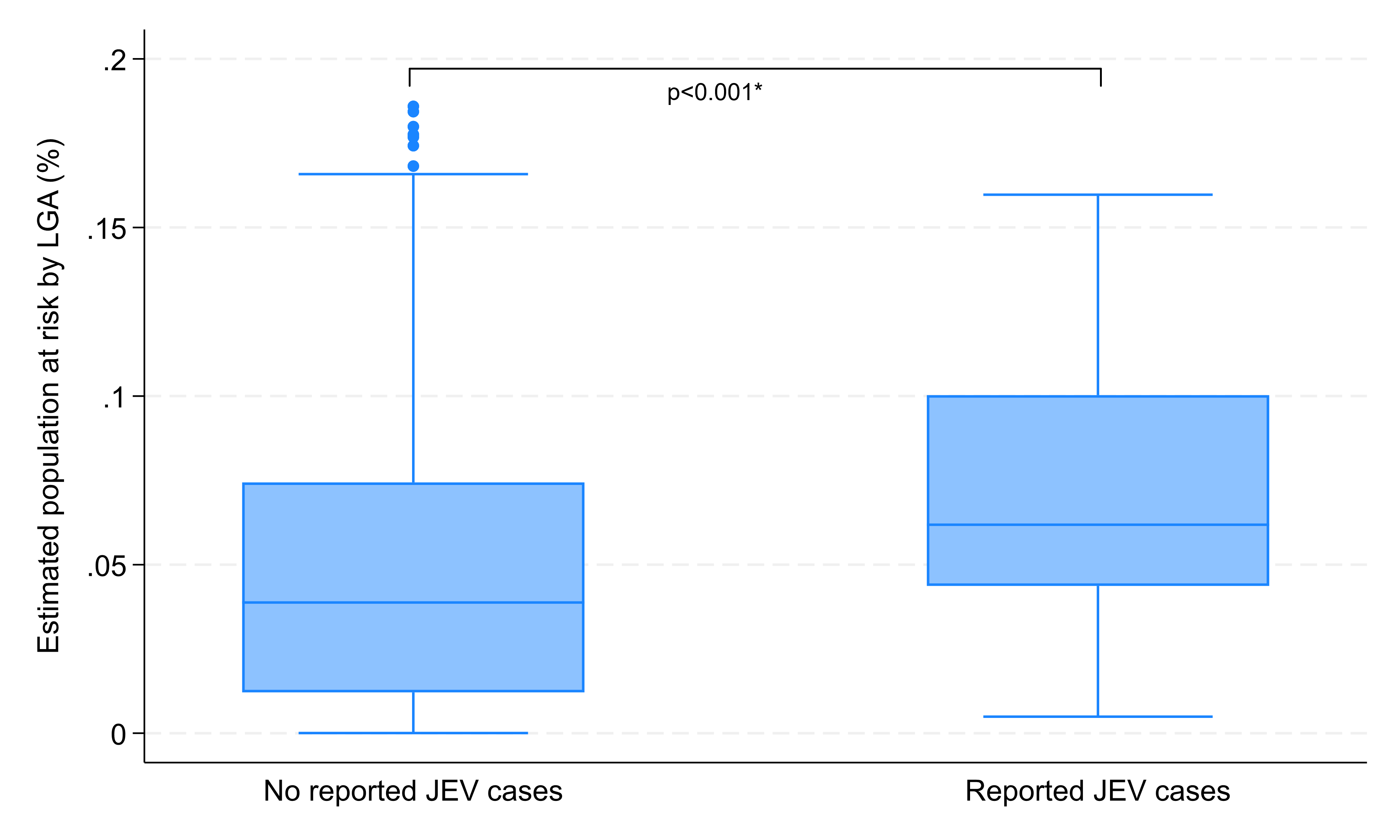

Supplement: S5 Fig — (TIF) [file pntd.0013722.s008.tif]
