# Supplementary material for: Ecological suitability of Japanese encephalitis virus in Australia: A modelling analysis of vector-host transmission dynamics to potential spillover in humans
Source: PLoS Negl Trop Dis. 2025 Nov 17;19(11):e0013722. doi: 10.1371/journal.pntd.0013722 (PMC12654935; doi:10.1371/journal.pntd.0013722)
Supplement: S3 Table — (DOCX) [file pntd.0013722.s007.docx]

**S7 Table: Potential human population at risk of JEV infection from spillover by LGA with count and percent at risk, including 95% uncertainty interval (UIs)**

| **LGA name** | **State name** | **Total population** | **Population at risk (count)** | **95% L** | **95% U** | **% pop at risk** | **95% L** | **95% U** |
| --- | --- | --- | --- | --- | --- | --- | --- | --- |
| Willoughby | New South Wales | 72,959 | 13,565 | 457 | 50,699 | 18.6% | 0.6% | 69.5% |
| Camden | New South Wales | 126,276 | 23,281 | 802 | 96,979 | 18.4% | 0.6% | 76.8% |
| Randwick | New South Wales | 134,303 | 24,163 | 852 | 89,001 | 18.0% | 0.6% | 66.3% |
| Liverpool | New South Wales | 242,230 | 43,015 | 1,470 | 172,730 | 17.8% | 0.6% | 71.3% |
| Blacktown | New South Wales | 407,880 | 72,118 | 2,620 | 309,316 | 17.7% | 0.6% | 75.8% |
| Penrith | New South Wales | 220,137 | 38,360 | 1,376 | 157,826 | 17.4% | 0.6% | 71.7% |
| Campbelltown | New South Wales | 179,405 | 30,178 | 1,015 | 119,248 | 16.8% | 0.6% | 66.5% |
| Bayside | New South Wales | 165,584 | 27,457 | 963 | 104,491 | 16.6% | 0.6% | 63.1% |
| Cumberland | New South Wales | 243,047 | 39,902 | 1,336 | 151,276 | 16.4% | 0.5% | 62.2% |
| Inner West | New South Wales | 186,042 | 30,400 | 1,050 | 113,861 | 16.3% | 0.6% | 61.2% |
| Fairfield | New South Wales | 206,847 | 33,266 | 1,125 | 128,375 | 16.1% | 0.5% | 62.1% |
| Parramatta | New South Wales | 249,084 | 40,046 | 1,352 | 156,910 | 16.1% | 0.5% | 63.0% |
| Wodonga | Victoria | 43,729 | 6,985 | 251 | 29,910 | 16.0% | 0.6% | 68.4% |
| Lane Cove | New South Wales | 34,367 | 5,432 | 188 | 20,573 | 15.8% | 0.5% | 59.9% |
| Canterbury-Bankstown | New South Wales | 371,133 | 57,682 | 1,952 | 222,454 | 15.5% | 0.5% | 59.9% |
| Ryde | New South Wales | 129,110 | 19,976 | 671 | 75,703 | 15.5% | 0.5% | 58.6% |
| Ku-ring-gai | New South Wales | 122,614 | 18,800 | 618 | 72,989 | 15.3% | 0.5% | 59.5% |
| Sydney | New South Wales | 211,361 | 32,091 | 1,124 | 123,295 | 15.2% | 0.5% | 58.3% |
| North Sydney | New South Wales | 70,895 | 10,619 | 373 | 40,240 | 15.0% | 0.5% | 56.8% |
| Hawkesbury | New South Wales | 68,403 | 10,160 | 355 | 40,678 | 14.9% | 0.5% | 59.5% |
| Maitland | New South Wales | 93,810 | 13,879 | 455 | 55,928 | 14.8% | 0.5% | 59.6% |
| Port Macquarie-Hastings | New South Wales | 86,822 | 12,824 | 445 | 49,229 | 14.8% | 0.5% | 56.7% |
| Melbourne | Victoria | 161,538 | 23,751 | 713 | 93,679 | 14.7% | 0.4% | 58.0% |
| Sutherland | New South Wales | 219,691 | 32,233 | 1,101 | 122,027 | 14.7% | 0.5% | 55.5% |
| Woollahra | New South Wales | 57,437 | 8,240 | 290 | 30,233 | 14.3% | 0.5% | 52.6% |
| Federation | New South Wales | 12,721 | 1,781 | 61 | 7,242 | 14.0% | 0.5% | 56.9% |
| Kempsey | New South Wales | 30,994 | 4,294 | 155 | 16,774 | 13.9% | 0.5% | 54.1% |
| Dungog | New South Wales | 9,639 | 1,302 | 44 | 5,238 | 13.5% | 0.5% | 54.3% |
| Clarence Valley | New South Wales | 54,198 | 7,262 | 270 | 28,733 | 13.4% | 0.5% | 53.0% |
| Wollondilly | New South Wales | 56,198 | 7,388 | 244 | 29,724 | 13.1% | 0.4% | 52.9% |
| Moira | Victoria | 30,564 | 3,994 | 133 | 16,775 | 13.1% | 0.4% | 54.9% |
| Cessnock | New South Wales | 65,091 | 8,435 | 278 | 34,608 | 13.0% | 0.4% | 53.2% |
| Berrigan | New South Wales | 8,449 | 1,089 | 38 | 4,831 | 12.9% | 0.5% | 57.2% |
| Boroondara | Victoria | 162,694 | 20,830 | 661 | 81,185 | 12.8% | 0.4% | 49.9% |
| Belyuen | Northern Territory | 165 | 21 | 1 | 88 | 12.8% | 0.5% | 53.1% |
| Greater Shepparton | Victoria | 68,934 | 8,784 | 279 | 36,717 | 12.7% | 0.4% | 53.3% |
| Wangaratta | Victoria | 29,884 | 3,803 | 141 | 16,652 | 12.7% | 0.5% | 55.7% |
| Wagga Wagga | New South Wales | 68,260 | 8,655 | 241 | 33,902 | 12.7% | 0.4% | 49.7% |
| Lismore | New South Wales | 44,317 | 5,616 | 222 | 24,646 | 12.7% | 0.5% | 55.6% |
| East Fremantle | Western Australia | 5,601 | 706 | 11 | 2,991 | 12.6% | 0.2% | 53.4% |
| Shoalhaven | New South Wales | 107,480 | 13,530 | 442 | 53,630 | 12.6% | 0.4% | 49.9% |
| The Hills | New South Wales | 203,845 | 25,113 | 893 | 112,924 | 12.3% | 0.4% | 55.4% |
| Eurobodalla | New South Wales | 37,514 | 4,584 | 138 | 18,651 | 12.2% | 0.4% | 49.7% |
| Latrobe (Vic.) | Victoria | 77,388 | 9,304 | 317 | 36,059 | 12.0% | 0.4% | 46.6% |
| Moonee Valley | Victoria | 123,979 | 14,830 | 449 | 57,625 | 12.0% | 0.4% | 46.5% |
| Mid-Coast | New South Wales | 94,566 | 11,240 | 391 | 44,566 | 11.9% | 0.4% | 47.1% |
| Port Phillip | Victoria | 98,773 | 11,663 | 352 | 45,912 | 11.8% | 0.4% | 46.5% |
| Kwinana | Western Australia | 49,079 | 5,766 | 131 | 23,590 | 11.7% | 0.3% | 48.1% |
| Brisbane | Queensland | 1,266,544 | 148,180 | 5,724 | 593,830 | 11.7% | 0.5% | 46.9% |
| Port Stephens | New South Wales | 71,608 | 8,357 | 285 | 33,231 | 11.7% | 0.4% | 46.4% |
| Canada Bay | New South Wales | 77,786 | 9,065 | 308 | 34,037 | 11.7% | 0.4% | 43.8% |
| Redland | Queensland | 158,597 | 18,059 | 675 | 71,000 | 11.4% | 0.4% | 44.8% |
| Albury | New South Wales | 56,479 | 6,430 | 205 | 26,267 | 11.4% | 0.4% | 46.5% |
| Claremont | Western Australia | 10,687 | 1,195 | 18 | 5,060 | 11.2% | 0.2% | 47.4% |
| Perth | Western Australia | 24,042 | 2,688 | 47 | 11,352 | 11.2% | 0.2% | 47.2% |
| Cockburn | Western Australia | 118,715 | 13,265 | 261 | 54,901 | 11.2% | 0.2% | 46.2% |
| Palm Island | Queensland | 2,077 | 226 | 9 | 927 | 10.9% | 0.4% | 44.6% |
| Kiama | New South Wales | 21,014 | 2,286 | 77 | 8,865 | 10.9% | 0.4% | 42.2% |
| Burwood | New South Wales | 43,359 | 4,669 | 159 | 18,730 | 10.8% | 0.4% | 43.2% |
| Darebin | Victoria | 149,124 | 15,997 | 514 | 63,440 | 10.7% | 0.3% | 42.5% |
| Rockingham | Western Australia | 140,448 | 14,928 | 336 | 61,333 | 10.6% | 0.2% | 43.7% |
| Bellingen | New South Wales | 13,239 | 1,405 | 52 | 5,554 | 10.6% | 0.4% | 42.0% |
| Yarra | Victoria | 103,012 | 10,915 | 328 | 42,659 | 10.6% | 0.3% | 41.4% |
| Logan | Queensland | 366,017 | 38,755 | 1,494 | 159,713 | 10.6% | 0.4% | 43.6% |
| Maribyrnong | Victoria | 89,916 | 9,335 | 269 | 36,801 | 10.4% | 0.3% | 40.9% |
| Litchfield | Northern Territory | 23,079 | 2,393 | 103 | 10,406 | 10.4% | 0.4% | 45.1% |
| Cook | Queensland | 5,059 | 519 | 20 | 2,171 | 10.3% | 0.4% | 42.9% |
| Hunters Hill | New South Wales | 13,713 | 1,407 | 49 | 5,364 | 10.3% | 0.4% | 39.1% |
| Rockhampton | Queensland | 83,406 | 8,495 | 362 | 32,604 | 10.2% | 0.4% | 39.1% |
| South Perth | Western Australia | 38,910 | 3,952 | 72 | 16,613 | 10.2% | 0.2% | 42.7% |
| Mitcham | South Australia | 68,055 | 6,873 | 108 | 28,996 | 10.1% | 0.2% | 42.6% |
| Banyule | Victoria | 124,546 | 12,567 | 425 | 50,208 | 10.1% | 0.3% | 40.3% |
| Richmond Valley | New South Wales | 23,243 | 2,340 | 90 | 9,335 | 10.1% | 0.4% | 40.2% |
| Towong | Victoria | 6,165 | 620 | 24 | 2,519 | 10.1% | 0.4% | 40.9% |
| Lockhart | New South Wales | 3,318 | 333 | 9 | 1,336 | 10.0% | 0.3% | 40.3% |
| Melville | Western Australia | 103,605 | 10,363 | 170 | 43,628 | 10.0% | 0.2% | 42.1% |
| Mosman | New South Wales | 26,284 | 2,624 | 91 | 9,890 | 10.0% | 0.3% | 37.6% |
| Murray | Western Australia | 19,304 | 1,926 | 44 | 7,960 | 10.0% | 0.2% | 41.2% |
| Georges River | New South Wales | 148,258 | 14,785 | 510 | 58,830 | 10.0% | 0.3% | 39.7% |
| Gosnells | Western Australia | 131,031 | 13,034 | 249 | 54,336 | 9.9% | 0.2% | 41.5% |
| Victoria Park | Western Australia | 40,010 | 3,970 | 65 | 16,697 | 9.9% | 0.2% | 41.7% |
| Strathfield | New South Wales | 41,147 | 4,069 | 139 | 17,478 | 9.9% | 0.3% | 42.5% |
| Ballina | New South Wales | 43,778 | 4,322 | 162 | 17,120 | 9.9% | 0.4% | 39.1% |
| Ipswich | Queensland | 240,485 | 23,580 | 981 | 99,952 | 9.8% | 0.4% | 41.6% |
| Indigo | Victoria | 17,741 | 1,733 | 64 | 7,378 | 9.8% | 0.4% | 41.6% |
| Casey | Victoria | 374,860 | 36,614 | 1,196 | 147,836 | 9.8% | 0.3% | 39.4% |
| Strathbogie | Victoria | 11,390 | 1,103 | 38 | 4,717 | 9.7% | 0.3% | 41.4% |
| Stonnington | Victoria | 103,771 | 10,036 | 309 | 40,388 | 9.7% | 0.3% | 38.9% |
| Campaspe | Victoria | 38,477 | 3,720 | 135 | 16,672 | 9.7% | 0.4% | 43.3% |
| Nillumbik | Victoria | 65,212 | 6,213 | 234 | 29,581 | 9.5% | 0.4% | 45.4% |
| Bundaberg | Queensland | 101,137 | 9,604 | 384 | 38,710 | 9.5% | 0.4% | 38.3% |
| Greater Bendigo | Victoria | 122,511 | 11,592 | 361 | 52,012 | 9.5% | 0.3% | 42.5% |
| Mosman Park | Western Australia | 8,006 | 753 | 13 | 3,200 | 9.4% | 0.2% | 40.0% |
| Shellharbour | New South Wales | 77,169 | 7,171 | 243 | 29,029 | 9.3% | 0.3% | 37.6% |
| Hornsby | New South Wales | 152,912 | 14,207 | 460 | 60,337 | 9.3% | 0.3% | 39.5% |
| Bassendean | Western Australia | 15,399 | 1,423 | 23 | 6,058 | 9.2% | 0.2% | 39.3% |
| Wellington | Victoria | 44,975 | 4,156 | 149 | 17,070 | 9.2% | 0.3% | 38.0% |
| Singleton | New South Wales | 25,701 | 2,370 | 88 | 9,782 | 9.2% | 0.3% | 38.1% |
| Blue Mountains | New South Wales | 78,449 | 7,211 | 242 | 30,203 | 9.2% | 0.3% | 38.5% |
| Armadale | Western Australia | 99,109 | 9,089 | 189 | 37,842 | 9.2% | 0.2% | 38.2% |
| Benalla | Victoria | 14,412 | 1,318 | 44 | 5,458 | 9.1% | 0.3% | 37.9% |
| Burdekin | Queensland | 16,855 | 1,527 | 63 | 6,209 | 9.1% | 0.4% | 36.8% |
| Wentworth | New South Wales | 7,439 | 662 | 18 | 2,630 | 8.9% | 0.2% | 35.4% |
| Coomalie | Northern Territory | 1,468 | 130 | 7 | 569 | 8.9% | 0.5% | 38.8% |
| East Gippsland | Victoria | 49,177 | 4,329 | 149 | 17,647 | 8.8% | 0.3% | 35.9% |
| Mornington | Queensland | 782 | 69 | 2 | 280 | 8.8% | 0.3% | 35.8% |
| Manningham | Victoria | 127,295 | 11,100 | 389 | 45,043 | 8.7% | 0.3% | 35.4% |
| Burnside | South Australia | 45,312 | 3,833 | 55 | 16,562 | 8.5% | 0.1% | 36.6% |
| Adelaide | South Australia | 27,960 | 2,365 | 34 | 10,069 | 8.5% | 0.1% | 36.0% |
| Capel | Western Australia | 18,597 | 1,572 | 33 | 6,710 | 8.5% | 0.2% | 36.1% |
| Gold Coast | Queensland | 625,623 | 52,462 | 1,949 | 205,874 | 8.4% | 0.3% | 32.9% |
| West Daly | Northern Territory | 3,423 | 284 | 16 | 1,222 | 8.3% | 0.5% | 35.7% |
| Whitehorse | Victoria | 172,598 | 14,245 | 470 | 58,238 | 8.3% | 0.3% | 33.7% |
| Greater Dandenong | Victoria | 163,458 | 13,436 | 415 | 56,237 | 8.2% | 0.3% | 34.4% |
| Swan | Western Australia | 164,239 | 13,500 | 263 | 56,513 | 8.2% | 0.2% | 34.4% |
| Coffs Harbour | New South Wales | 76,727 | 6,277 | 229 | 24,019 | 8.2% | 0.3% | 31.3% |
| Noosa | Queensland | 56,834 | 4,629 | 179 | 19,964 | 8.1% | 0.3% | 35.1% |
| Subiaco | Western Australia | 11,690 | 950 | 16 | 4,221 | 8.1% | 0.1% | 36.1% |
| Fraser Coast | Queensland | 109,240 | 8,858 | 355 | 39,077 | 8.1% | 0.3% | 35.8% |
| Waverley | New South Wales | 56,444 | 4,567 | 163 | 18,784 | 8.1% | 0.3% | 33.3% |
| Loddon | Victoria | 7,771 | 627 | 21 | 2,729 | 8.1% | 0.3% | 35.1% |
| Hume | Victoria | 254,800 | 20,512 | 734 | 95,261 | 8.1% | 0.3% | 37.4% |
| Yarra Ranges | Victoria | 156,808 | 12,616 | 493 | 51,308 | 8.0% | 0.3% | 32.7% |
| Greater Hume | New South Wales | 11,290 | 906 | 28 | 3,832 | 8.0% | 0.2% | 33.9% |
| Gladstone | Queensland | 64,864 | 5,203 | 205 | 22,757 | 8.0% | 0.3% | 35.1% |
| Vincent | Western Australia | 41,826 | 3,352 | 50 | 14,243 | 8.0% | 0.1% | 34.1% |
| Nambucca Valley | New South Wales | 20,309 | 1,612 | 61 | 6,677 | 7.9% | 0.3% | 32.9% |
| Muswellbrook | New South Wales | 16,552 | 1,299 | 48 | 5,310 | 7.8% | 0.3% | 32.1% |
| Yass Valley | New South Wales | 17,378 | 1,359 | 41 | 5,621 | 7.8% | 0.2% | 32.3% |
| Baw Baw | Victoria | 59,354 | 4,619 | 176 | 18,424 | 7.8% | 0.3% | 31.0% |
| Wollongong | New South Wales | 211,619 | 16,423 | 545 | 62,637 | 7.8% | 0.3% | 29.6% |
| Tea Tree Gully | South Australia | 98,252 | 7,609 | 126 | 32,419 | 7.7% | 0.1% | 33.0% |
| Townsville | Queensland | 196,144 | 14,860 | 564 | 65,525 | 7.6% | 0.3% | 33.4% |
| Northern Beaches | New South Wales | 244,488 | 18,387 | 629 | 69,063 | 7.5% | 0.3% | 28.2% |
| Cardinia | Victoria | 123,788 | 9,306 | 349 | 39,953 | 7.5% | 0.3% | 32.3% |
| Knox | Victoria | 164,455 | 12,361 | 430 | 51,452 | 7.5% | 0.3% | 31.3% |
| Moreton Bay | Queensland | 479,842 | 35,902 | 1,373 | 157,654 | 7.5% | 0.3% | 32.9% |
| Belmont | Western Australia | 43,360 | 3,231 | 54 | 13,661 | 7.5% | 0.1% | 31.5% |
| Palmerston | Northern Territory | 40,577 | 3,020 | 120 | 14,639 | 7.4% | 0.3% | 36.1% |
| Canning | Western Australia | 105,104 | 7,818 | 132 | 33,277 | 7.4% | 0.1% | 31.7% |
| Kingston (Vic.) | Victoria | 153,818 | 11,440 | 317 | 47,250 | 7.4% | 0.2% | 30.7% |
| Greater Geelong | Victoria | 275,105 | 20,444 | 364 | 87,184 | 7.4% | 0.1% | 31.7% |
| Murrindindi | Victoria | 15,238 | 1,103 | 45 | 4,548 | 7.2% | 0.3% | 29.8% |
| Mildura | Victoria | 57,264 | 4,124 | 106 | 16,506 | 7.2% | 0.2% | 28.8% |
| Murray River | New South Wales | 12,823 | 914 | 35 | 4,017 | 7.1% | 0.3% | 31.3% |
| Douglas | Queensland | 11,243 | 801 | 32 | 3,858 | 7.1% | 0.3% | 34.3% |
| Mitchell | Victoria | 51,741 | 3,678 | 125 | 15,055 | 7.1% | 0.2% | 29.1% |
| Gunnedah | New South Wales | 13,212 | 938 | 37 | 4,033 | 7.1% | 0.3% | 30.5% |
| Cambridge | Western Australia | 32,611 | 2,280 | 44 | 9,531 | 7.0% | 0.1% | 29.2% |
| Stirling | Western Australia | 229,345 | 15,917 | 242 | 68,302 | 6.9% | 0.1% | 29.8% |
| Wyndham | Victoria | 308,150 | 21,351 | 557 | 89,170 | 6.9% | 0.2% | 28.9% |
| Gannawarra | Victoria | 10,666 | 736 | 29 | 3,114 | 6.9% | 0.3% | 29.2% |
| Port Pirie | South Australia | 17,452 | 1,201 | 28 | 4,894 | 6.9% | 0.2% | 28.0% |
| Waroona | Western Australia | 4,345 | 297 | 7 | 1,263 | 6.8% | 0.2% | 29.1% |
| Frankston | Victoria | 142,212 | 9,681 | 274 | 44,274 | 6.8% | 0.2% | 31.1% |
| Golden Plains | Victoria | 25,237 | 1,714 | 56 | 7,219 | 6.8% | 0.2% | 28.6% |
| Harvey | Western Australia | 29,880 | 2,023 | 42 | 8,563 | 6.8% | 0.1% | 28.7% |
| Somerset | Queensland | 25,872 | 1,741 | 73 | 7,596 | 6.7% | 0.3% | 29.4% |
| Snowy Valleys | New South Wales | 14,901 | 995 | 43 | 4,105 | 6.7% | 0.3% | 27.5% |
| Whitsunday | Queensland | 36,593 | 2,435 | 98 | 11,005 | 6.7% | 0.3% | 30.1% |
| Byron | New South Wales | 35,835 | 2,381 | 91 | 9,733 | 6.6% | 0.3% | 27.2% |
| Mandurah | Western Australia | 91,299 | 6,001 | 144 | 24,398 | 6.6% | 0.2% | 26.7% |
| Adelaide Hills | South Australia | 43,672 | 2,868 | 72 | 13,023 | 6.6% | 0.2% | 29.8% |
| Wanneroo | Western Australia | 220,766 | 14,475 | 299 | 60,109 | 6.6% | 0.1% | 27.2% |
| Campbelltown (SA) | South Australia | 55,207 | 3,603 | 57 | 15,612 | 6.5% | 0.1% | 28.3% |
| Cairns | Queensland | 168,298 | 10,922 | 431 | 52,434 | 6.5% | 0.3% | 31.2% |
| Nedlands | Western Australia | 28,606 | 1,842 | 29 | 7,833 | 6.4% | 0.1% | 27.4% |
| Bega Valley | New South Wales | 35,038 | 2,256 | 73 | 9,162 | 6.4% | 0.2% | 26.1% |
| Forbes | New South Wales | 9,386 | 604 | 18 | 2,635 | 6.4% | 0.2% | 28.1% |
| Woorabinda | Queensland | 1,088 | 70 | 3 | 277 | 6.4% | 0.3% | 25.5% |
| Southern Grampians | Victoria | 16,395 | 1,050 | 40 | 4,177 | 6.4% | 0.2% | 25.5% |
| Maroondah | Victoria | 111,216 | 7,083 | 254 | 30,114 | 6.4% | 0.2% | 27.1% |
| Yarrabah | Queensland | 2,208 | 139 | 5 | 688 | 6.3% | 0.2% | 31.2% |
| Sunshine Coast | Queensland | 346,946 | 21,827 | 828 | 92,785 | 6.3% | 0.2% | 26.7% |
| Gingin | Western Australia | 5,884 | 370 | 8 | 1,598 | 6.3% | 0.1% | 27.2% |
| Narrabri | New South Wales | 12,672 | 792 | 34 | 3,214 | 6.3% | 0.3% | 25.4% |
| Alpine | Victoria | 13,185 | 820 | 40 | 3,589 | 6.2% | 0.3% | 27.2% |
| Monash | Victoria | 196,897 | 12,094 | 375 | 51,794 | 6.1% | 0.2% | 26.3% |
| Joondalup | Western Australia | 163,693 | 9,984 | 174 | 43,436 | 6.1% | 0.1% | 26.5% |
| Lockyer Valley | Queensland | 42,910 | 2,615 | 112 | 12,346 | 6.1% | 0.3% | 28.8% |
| Kowanyama | Queensland | 1,143 | 70 | 3 | 270 | 6.1% | 0.3% | 23.6% |
| Bass Coast | Victoria | 39,925 | 2,415 | 49 | 10,271 | 6.0% | 0.1% | 25.7% |
| Colac Otway | Victoria | 21,548 | 1,300 | 49 | 5,171 | 6.0% | 0.2% | 24.0% |
| Kyogle | New South Wales | 9,265 | 558 | 22 | 2,362 | 6.0% | 0.2% | 25.5% |
| Lake Macquarie | New South Wales | 199,574 | 11,958 | 404 | 47,759 | 6.0% | 0.2% | 23.9% |
| Burke | Queensland | 443 | 26 | 1 | 100 | 5.9% | 0.2% | 22.5% |
| Playford | South Australia | 104,512 | 6,150 | 105 | 26,396 | 5.9% | 0.1% | 25.3% |
| Glenelg | Victoria | 19,564 | 1,150 | 25 | 5,075 | 5.9% | 0.1% | 25.9% |
| Upper Hunter | New South Wales | 14,258 | 836 | 30 | 3,512 | 5.9% | 0.2% | 24.6% |
| Wingecarribee | New South Wales | 53,067 | 3,110 | 96 | 13,486 | 5.9% | 0.2% | 25.4% |
| Whittlesea | Victoria | 235,775 | 13,701 | 464 | 58,180 | 5.8% | 0.2% | 24.7% |
| Goondiwindi | Queensland | 10,609 | 615 | 30 | 2,804 | 5.8% | 0.3% | 26.4% |
| Adelaide Plains | South Australia | 10,170 | 589 | 14 | 2,795 | 5.8% | 0.1% | 27.5% |
| Merri-bek | Victoria | 162,456 | 9,398 | 304 | 42,598 | 5.8% | 0.2% | 26.2% |
| Edward River | New South Wales | 8,486 | 491 | 18 | 2,034 | 5.8% | 0.2% | 24.0% |
| Glen Eira | Victoria | 148,854 | 8,582 | 254 | 37,405 | 5.8% | 0.2% | 25.1% |
| Salisbury | South Australia | 146,698 | 8,405 | 154 | 35,938 | 5.7% | 0.1% | 24.5% |
| Wujal Wujal | Queensland | 289 | 17 | 1 | 93 | 5.7% | 0.2% | 32.3% |
| Livingstone | Queensland | 37,898 | 2,161 | 84 | 8,946 | 5.7% | 0.2% | 23.6% |
| Brimbank | Victoria | 191,885 | 10,917 | 364 | 50,381 | 5.7% | 0.2% | 26.3% |
| Corangamite | Victoria | 15,959 | 906 | 35 | 3,660 | 5.7% | 0.2% | 22.9% |
| Parkes | New South Wales | 14,301 | 809 | 22 | 3,467 | 5.7% | 0.2% | 24.2% |
| Prospect | South Australia | 17,842 | 1,004 | 15 | 4,478 | 5.6% | 0.1% | 25.1% |
| Ballarat | Victoria | 115,901 | 6,516 | 266 | 26,143 | 5.6% | 0.2% | 22.6% |
| Cootamundra-Gundagai | New South Wales | 11,390 | 637 | 21 | 2,681 | 5.6% | 0.2% | 23.5% |
| Barossa | South Australia | 26,915 | 1,494 | 33 | 6,409 | 5.6% | 0.1% | 23.8% |
| Darwin | Northern Territory | 81,088 | 4,485 | 171 | 18,631 | 5.5% | 0.2% | 23.0% |
| Newcastle | New South Wales | 160,248 | 8,718 | 298 | 35,499 | 5.4% | 0.2% | 22.2% |
| Hobsons Bay | Victoria | 86,776 | 4,700 | 129 | 19,556 | 5.4% | 0.1% | 22.5% |
| Northern Grampians | Victoria | 11,870 | 637 | 20 | 2,888 | 5.4% | 0.2% | 24.3% |
| Chittering | Western Australia | 6,248 | 335 | 6 | 1,449 | 5.4% | 0.1% | 23.2% |
| Tweed | New South Wales | 88,891 | 4,731 | 178 | 18,100 | 5.3% | 0.2% | 20.4% |
| Hope Vale | Queensland | 1,017 | 54 | 2 | 298 | 5.3% | 0.2% | 29.3% |
| Unincorporated ACT | Australian Capital Territory | 456,522 | 23,861 | 873 | 100,484 | 5.2% | 0.2% | 22.0% |
| Bayswater | Western Australia | 72,540 | 3,790 | 63 | 16,292 | 5.2% | 0.1% | 22.5% |
| Pyrenees | Victoria | 7,687 | 399 | 15 | 1,675 | 5.2% | 0.2% | 21.8% |
| Mackay | Queensland | 122,947 | 6,361 | 241 | 27,276 | 5.2% | 0.2% | 22.2% |
| Gympie | Queensland | 54,563 | 2,821 | 123 | 13,096 | 5.2% | 0.2% | 24.0% |
| Kalamunda | Western Australia | 61,335 | 3,170 | 65 | 13,866 | 5.2% | 0.1% | 22.6% |
| Lachlan | New South Wales | 6,113 | 314 | 11 | 1,269 | 5.1% | 0.2% | 20.8% |
| Hinchinbrook | Queensland | 10,813 | 553 | 22 | 2,712 | 5.1% | 0.2% | 25.1% |
| Cassowary Coast | Queensland | 27,573 | 1,403 | 55 | 6,959 | 5.1% | 0.2% | 25.2% |
| Tiwi Islands | Northern Territory | 2,385 | 121 | 5 | 545 | 5.1% | 0.2% | 22.9% |
| Moorabool | Victoria | 38,483 | 1,932 | 86 | 7,944 | 5.0% | 0.2% | 20.6% |
| Central Goldfields | Victoria | 13,502 | 674 | 21 | 2,931 | 5.0% | 0.2% | 21.7% |
| Melton | Victoria | 194,936 | 9,644 | 354 | 42,309 | 4.9% | 0.2% | 21.7% |
| North Burnett | Queensland | 10,221 | 505 | 23 | 2,345 | 4.9% | 0.2% | 22.9% |
| Murrumbidgee | New South Wales | 3,562 | 176 | 6 | 731 | 4.9% | 0.2% | 20.5% |
| Balranald | New South Wales | 2,246 | 111 | 3 | 451 | 4.9% | 0.2% | 20.1% |
| Hepburn | Victoria | 16,465 | 809 | 39 | 3,515 | 4.9% | 0.2% | 21.3% |
| Norwood Payneham and St Peters | South Australia | 36,357 | 1,779 | 27 | 7,875 | 4.9% | 0.1% | 21.7% |
| Scenic Rim | Queensland | 43,942 | 2,149 | 85 | 10,060 | 4.9% | 0.2% | 22.9% |
| Horsham | Victoria | 20,325 | 992 | 37 | 3,902 | 4.9% | 0.2% | 19.2% |
| Unincorporated NT | Northern Territory | 6,453 | 314 | 14 | 1,447 | 4.9% | 0.2% | 22.4% |
| West Wimmera | Victoria | 3,930 | 189 | 7 | 771 | 4.8% | 0.2% | 19.6% |
| Clare and Gilbert Valleys | South Australia | 9,404 | 451 | 12 | 2,258 | 4.8% | 0.1% | 24.0% |
| Moyne | Victoria | 17,763 | 848 | 22 | 3,612 | 4.8% | 0.1% | 20.3% |
| South Gippsland | Victoria | 30,524 | 1,447 | 36 | 6,266 | 4.7% | 0.1% | 20.5% |
| Tablelands | Queensland | 26,829 | 1,266 | 52 | 6,497 | 4.7% | 0.2% | 24.2% |
| Narrandera | New South Wales | 5,687 | 267 | 9 | 1,329 | 4.7% | 0.2% | 23.4% |
| Mount Barker | South Australia | 41,150 | 1,919 | 47 | 8,476 | 4.7% | 0.1% | 20.6% |
| Temora | New South Wales | 5,990 | 279 | 9 | 1,392 | 4.7% | 0.1% | 23.2% |
| Cowra | New South Wales | 12,738 | 588 | 20 | 2,428 | 4.6% | 0.2% | 19.1% |
| Warrnambool | Victoria | 35,166 | 1,624 | 29 | 7,088 | 4.6% | 0.1% | 20.2% |
| Cherbourg | Queensland | 1,049 | 48 | 2 | 241 | 4.6% | 0.2% | 23.0% |
| Bland | New South Wales | 5,471 | 251 | 6 | 1,051 | 4.6% | 0.1% | 19.2% |
| Weddin | New South Wales | 3,598 | 164 | 5 | 720 | 4.6% | 0.1% | 20.0% |
| Swan Hill | Victoria | 21,138 | 945 | 35 | 3,772 | 4.5% | 0.2% | 17.8% |
| Narromine | New South Wales | 6,441 | 287 | 11 | 1,457 | 4.5% | 0.2% | 22.6% |
| Northern Peninsula Area | Queensland | 2,862 | 128 | 6 | 629 | 4.5% | 0.2% | 22.0% |
| Manjimup | Western Australia | 9,430 | 420 | 10 | 1,790 | 4.4% | 0.1% | 19.0% |
| Mount Alexander | Victoria | 20,292 | 895 | 34 | 4,002 | 4.4% | 0.2% | 19.7% |
| Lower Eyre Peninsula | South Australia | 5,640 | 249 | 2 | 1,124 | 4.4% | 0.0% | 19.9% |
| Lockhart River | Queensland | 659 | 29 | 1 | 177 | 4.4% | 0.2% | 26.9% |
| Macedon Ranges | Victoria | 52,060 | 2,275 | 101 | 9,635 | 4.4% | 0.2% | 18.5% |
| Gwydir | New South Wales | 4,899 | 212 | 7 | 1,004 | 4.3% | 0.1% | 20.5% |
| Port Augusta | South Australia | 13,929 | 599 | 17 | 2,351 | 4.3% | 0.1% | 16.9% |
| Moree Plains | New South Wales | 12,587 | 540 | 24 | 2,272 | 4.3% | 0.2% | 18.0% |
| Naracoorte Lucindale | South Australia | 8,925 | 376 | 14 | 1,531 | 4.2% | 0.2% | 17.2% |
| Dubbo | New South Wales | 55,849 | 2,347 | 77 | 10,399 | 4.2% | 0.1% | 18.6% |
| Mount Gambier | South Australia | 26,890 | 1,125 | 28 | 5,130 | 4.2% | 0.1% | 19.1% |
| Balonne | Queensland | 4,378 | 183 | 9 | 705 | 4.2% | 0.2% | 16.1% |
| Wyndham-East Kimberley | Western Australia | 8,058 | 336 | 15 | 1,294 | 4.2% | 0.2% | 16.1% |
| Warren | New South Wales | 2,603 | 108 | 4 | 458 | 4.2% | 0.1% | 17.6% |
| Serpentine-Jarrahdale | Western Australia | 36,184 | 1,498 | 39 | 6,944 | 4.1% | 0.1% | 19.2% |
| Western Downs | Queensland | 34,583 | 1,425 | 67 | 6,874 | 4.1% | 0.2% | 19.9% |
| Dardanup | Western Australia | 15,468 | 636 | 12 | 2,756 | 4.1% | 0.1% | 17.8% |
| Albany | Western Australia | 39,193 | 1,598 | 22 | 7,307 | 4.1% | 0.1% | 18.6% |
| Unley | South Australia | 44,238 | 1,804 | 27 | 8,018 | 4.1% | 0.1% | 18.1% |
| Moora | Western Australia | 2,371 | 96 | 1 | 415 | 4.0% | 0.1% | 17.5% |
| Bayside (Vic.) | Victoria | 104,297 | 4,196 | 121 | 19,191 | 4.0% | 0.1% | 18.4% |
| Goulburn Mulwaree | New South Wales | 32,374 | 1,295 | 47 | 5,493 | 4.0% | 0.1% | 17.0% |
| Mid-Western | New South Wales | 25,786 | 1,028 | 35 | 4,585 | 4.0% | 0.1% | 17.8% |
| Coorong | South Australia | 5,224 | 208 | 6 | 975 | 4.0% | 0.1% | 18.7% |
| West Torrens | South Australia | 55,392 | 2,203 | 35 | 9,705 | 4.0% | 0.1% | 17.5% |
| Leeton | New South Wales | 11,485 | 454 | 14 | 1,884 | 4.0% | 0.1% | 16.4% |
| Kingston (SA) | South Australia | 2,076 | 82 | 2 | 354 | 3.9% | 0.1% | 17.0% |
| West Arnhem | Northern Territory | 6,680 | 262 | 13 | 1,231 | 3.9% | 0.2% | 18.4% |
| Fremantle | Western Australia | 35,775 | 1,401 | 25 | 6,313 | 3.9% | 0.1% | 17.6% |
| Tumby Bay | South Australia | 2,754 | 108 | 2 | 463 | 3.9% | 0.1% | 16.8% |
| Copper Coast | South Australia | 15,275 | 593 | 10 | 2,509 | 3.9% | 0.1% | 16.4% |
| Toodyay | Western Australia | 4,783 | 185 | 3 | 812 | 3.9% | 0.1% | 17.0% |
| Brewarrina | New South Wales | 1,416 | 54 | 2 | 212 | 3.8% | 0.2% | 15.0% |
| Onkaparinga | South Australia | 173,773 | 6,605 | 115 | 28,361 | 3.8% | 0.1% | 16.3% |
| Tamworth | New South Wales | 64,524 | 2,433 | 98 | 10,341 | 3.8% | 0.2% | 16.0% |
| Mansfield | Victoria | 10,308 | 388 | 19 | 1,714 | 3.8% | 0.2% | 16.6% |
| Light | South Australia | 17,156 | 635 | 15 | 3,054 | 3.7% | 0.1% | 17.8% |
| Central Highlands (Qld) | Queensland | 28,568 | 1,046 | 48 | 3,928 | 3.7% | 0.2% | 13.8% |
| Wattle Range | South Australia | 12,111 | 433 | 13 | 1,833 | 3.6% | 0.1% | 15.1% |
| Southern Downs | Queensland | 36,940 | 1,318 | 56 | 6,728 | 3.6% | 0.2% | 18.2% |
| Gilgandra | New South Wales | 4,261 | 152 | 5 | 664 | 3.6% | 0.1% | 15.6% |
| Hilltops | New South Wales | 19,211 | 684 | 25 | 3,297 | 3.6% | 0.1% | 17.2% |
| Renmark Paringa | South Australia | 10,034 | 353 | 13 | 1,353 | 3.5% | 0.1% | 13.5% |
| Hay | New South Wales | 2,828 | 99 | 3 | 410 | 3.5% | 0.1% | 14.5% |
| Augusta Margaret River | Western Australia | 17,421 | 604 | 14 | 2,812 | 3.5% | 0.1% | 16.1% |
| Ararat | Victoria | 11,711 | 406 | 15 | 1,738 | 3.5% | 0.1% | 14.8% |
| Port Lincoln | South Australia | 14,782 | 502 | 3 | 2,269 | 3.4% | 0.0% | 15.4% |
| Mornington Peninsula | Victoria | 164,046 | 5,545 | 129 | 24,451 | 3.4% | 0.1% | 14.9% |
| Port Adelaide Enfield | South Australia | 131,446 | 4,437 | 66 | 19,665 | 3.4% | 0.1% | 15.0% |
| Wongan-Ballidu | Western Australia | 1,338 | 45 | 1 | 188 | 3.4% | 0.1% | 14.0% |
| Isaac | Queensland | 22,741 | 766 | 28 | 3,076 | 3.4% | 0.1% | 13.5% |
| Carpentaria | Queensland | 2,177 | 71 | 3 | 268 | 3.3% | 0.1% | 12.3% |
| Plantagenet | Western Australia | 5,587 | 179 | 3 | 800 | 3.2% | 0.1% | 14.3% |
| South Burnett | Queensland | 33,952 | 1,089 | 49 | 6,279 | 3.2% | 0.1% | 18.5% |
| Mareeba | Queensland | 23,386 | 749 | 32 | 3,741 | 3.2% | 0.1% | 16.0% |
| Walgett | New South Wales | 5,554 | 177 | 7 | 745 | 3.2% | 0.1% | 13.4% |
| Banana | Queensland | 14,795 | 471 | 21 | 2,125 | 3.2% | 0.1% | 14.4% |
| Toowoomba | Queensland | 178,326 | 5,649 | 234 | 28,370 | 3.2% | 0.1% | 15.9% |
| Murray Bridge | South Australia | 22,604 | 716 | 26 | 3,453 | 3.2% | 0.1% | 15.3% |
| Liverpool Plains | New South Wales | 7,553 | 238 | 9 | 1,127 | 3.2% | 0.1% | 14.9% |
| Yarriambiack | Victoria | 6,409 | 201 | 8 | 801 | 3.1% | 0.1% | 12.5% |
| Junee | New South Wales | 6,463 | 199 | 6 | 886 | 3.1% | 0.1% | 13.7% |
| Busselton | Western Australia | 41,016 | 1,260 | 27 | 5,544 | 3.1% | 0.1% | 13.5% |
| Yorke Peninsula | South Australia | 10,505 | 315 | 4 | 1,378 | 3.0% | 0.0% | 13.1% |
| Warrumbungle | New South Wales | 9,252 | 278 | 10 | 1,451 | 3.0% | 0.1% | 15.7% |
| Northern Areas | South Australia | 4,666 | 140 | 2 | 604 | 3.0% | 0.0% | 13.0% |
| Hindmarsh | Victoria | 5,624 | 168 | 7 | 657 | 3.0% | 0.1% | 11.7% |
| Mundaring | Western Australia | 40,680 | 1,213 | 25 | 5,557 | 3.0% | 0.1% | 13.7% |
| Dandaragan | Western Australia | 3,466 | 102 | 3 | 430 | 2.9% | 0.1% | 12.4% |
| Cabonne | New South Wales | 14,093 | 415 | 14 | 1,980 | 2.9% | 0.1% | 14.0% |
| Wakefield | South Australia | 6,944 | 202 | 5 | 1,002 | 2.9% | 0.1% | 14.4% |
| Tenterfield | New South Wales | 7,124 | 207 | 9 | 911 | 2.9% | 0.1% | 12.8% |
| Queanbeyan-Palerang | New South Wales | 64,330 | 1,854 | 79 | 8,111 | 2.9% | 0.1% | 12.6% |
| Donnybrook-Balingup | Western Australia | 6,333 | 179 | 5 | 801 | 2.8% | 0.1% | 12.6% |
| Bourke | New South Wales | 2,351 | 67 | 3 | 265 | 2.8% | 0.1% | 11.3% |
| Coolamon | New South Wales | 4,468 | 126 | 4 | 582 | 2.8% | 0.1% | 13.0% |
| Loxton Waikerie | South Australia | 11,653 | 328 | 14 | 1,538 | 2.8% | 0.1% | 13.2% |
| Carrathool | New South Wales | 2,894 | 81 | 3 | 338 | 2.8% | 0.1% | 11.7% |
| Marion | South Australia | 101,557 | 2,833 | 43 | 12,533 | 2.8% | 0.0% | 12.3% |
| Franklin Harbour | South Australia | 1,229 | 33 | 1 | 140 | 2.7% | 0.1% | 11.4% |
| Alexandrina | South Australia | 29,213 | 792 | 15 | 3,695 | 2.7% | 0.1% | 12.6% |
| Mid Murray | South Australia | 9,438 | 251 | 10 | 1,127 | 2.7% | 0.1% | 11.9% |
| Walkerville | South Australia | 11,161 | 292 | 4 | 1,302 | 2.6% | 0.0% | 11.7% |
| Gawler | South Australia | 24,363 | 633 | 12 | 2,815 | 2.6% | 0.1% | 11.6% |
| East Arnhem | Northern Territory | 9,381 | 243 | 11 | 1,125 | 2.6% | 0.1% | 12.0% |
| Buloke | Victoria | 6,100 | 156 | 6 | 727 | 2.6% | 0.1% | 11.9% |
| Barunga West | South Australia | 2,115 | 54 | 1 | 230 | 2.6% | 0.1% | 10.9% |
| Orange | New South Wales | 43,604 | 1,106 | 36 | 4,997 | 2.5% | 0.1% | 11.5% |
| Cottesloe | Western Australia | 7,131 | 180 | 4 | 847 | 2.5% | 0.1% | 11.9% |
| Victor Harbor | South Australia | 15,635 | 393 | 4 | 1,832 | 2.5% | 0.0% | 11.7% |
| Cranbrook | Western Australia | 1,140 | 29 | 1 | 123 | 2.5% | 0.1% | 10.8% |
| Griffith | New South Wales | 26,899 | 673 | 22 | 2,862 | 2.5% | 0.1% | 10.6% |
| Grant | South Australia | 9,533 | 238 | 7 | 1,115 | 2.5% | 0.1% | 11.7% |
| Inverell | New South Wales | 18,077 | 451 | 21 | 2,136 | 2.5% | 0.1% | 11.8% |
| Irwin | Western Australia | 3,580 | 87 | 3 | 381 | 2.4% | 0.1% | 10.6% |
| Northam | Western Australia | 11,944 | 291 | 4 | 1,284 | 2.4% | 0.0% | 10.8% |
| Surf Coast | Victoria | 37,273 | 901 | 19 | 3,804 | 2.4% | 0.1% | 10.2% |
| Berri Barmera | South Australia | 10,998 | 266 | 11 | 1,032 | 2.4% | 0.1% | 9.4% |
| Victoria Daly | Northern Territory | 3,271 | 78 | 4 | 334 | 2.4% | 0.1% | 10.2% |
| Cobar | New South Wales | 4,046 | 95 | 3 | 372 | 2.3% | 0.1% | 9.2% |
| York | Western Australia | 3,566 | 81 | 1 | 376 | 2.3% | 0.0% | 10.6% |
| Mount Isa | Queensland | 19,111 | 430 | 12 | 1,721 | 2.3% | 0.1% | 9.0% |
| Kangaroo Island | South Australia | 4,366 | 97 | 1 | 438 | 2.2% | 0.0% | 10.0% |
| Katherine | Northern Territory | 10,770 | 239 | 13 | 965 | 2.2% | 0.1% | 9.0% |
| Barcaldine | Queensland | 2,864 | 64 | 2 | 260 | 2.2% | 0.1% | 9.1% |
| Paroo | Queensland | 1,722 | 38 | 2 | 145 | 2.2% | 0.1% | 8.4% |
| Denmark | Western Australia | 6,313 | 138 | 3 | 646 | 2.2% | 0.0% | 10.2% |
| Yankalilla | South Australia | 5,835 | 125 | 2 | 546 | 2.1% | 0.0% | 9.4% |
| Victoria Plains | Western Australia | 826 | 18 | 0 | 78 | 2.1% | 0.0% | 9.4% |
| Nannup | Western Australia | 1,589 | 34 | 1 | 164 | 2.1% | 0.1% | 10.3% |
| King Island | Tasmania | 1,643 | 34 | 1 | 160 | 2.1% | 0.0% | 9.7% |
| Unincorp. Other Territories | Other Territories | 2,721 | 57 | 2 | 220 | 2.1% | 0.1% | 8.1% |
| Esperance | Western Australia | 13,700 | 285 | 4 | 1,261 | 2.1% | 0.0% | 9.2% |
| Upper Lachlan | New South Wales | 8,547 | 178 | 7 | 812 | 2.1% | 0.1% | 9.5% |
| Tatiara | South Australia | 7,040 | 145 | 4 | 646 | 2.1% | 0.1% | 9.2% |
| Longreach | Queensland | 3,726 | 77 | 2 | 305 | 2.1% | 0.1% | 8.2% |
| Wagin | Western Australia | 1,817 | 36 | 1 | 156 | 2.0% | 0.0% | 8.6% |
| Bathurst | New South Wales | 44,094 | 882 | 38 | 3,852 | 2.0% | 0.1% | 8.7% |
| Lithgow | New South Wales | 20,815 | 408 | 16 | 1,984 | 2.0% | 0.1% | 9.5% |
| Collie | Western Australia | 9,143 | 176 | 5 | 788 | 1.9% | 0.1% | 8.6% |
| Katanning | Western Australia | 4,234 | 82 | 2 | 349 | 1.9% | 0.0% | 8.2% |
| Cloncurry | Queensland | 3,783 | 70 | 2 | 272 | 1.9% | 0.1% | 7.2% |
| Coonamble | New South Wales | 3,823 | 70 | 3 | 339 | 1.8% | 0.1% | 8.9% |
| Greater Geraldton | Western Australia | 38,772 | 710 | 21 | 2,985 | 1.8% | 0.1% | 7.7% |
| Central Coast (NSW) | New South Wales | 320,883 | 5,700 | 199 | 23,150 | 1.8% | 0.1% | 7.2% |
| Derby-West Kimberley | Western Australia | 8,464 | 150 | 5 | 563 | 1.8% | 0.1% | 6.6% |
| Broome | Western Australia | 18,101 | 320 | 14 | 1,159 | 1.8% | 0.1% | 6.4% |
| Snowy Monaro | New South Wales | 21,821 | 377 | 22 | 1,737 | 1.7% | 0.1% | 8.0% |
| Ravensthorpe | Western Australia | 1,947 | 34 | 1 | 147 | 1.7% | 0.0% | 7.5% |
| Launceston | Tasmania | 71,420 | 1,220 | 20 | 5,495 | 1.7% | 0.0% | 7.7% |
| Blackall Tambo | Queensland | 1,933 | 32 | 1 | 124 | 1.6% | 0.1% | 6.4% |
| Flinders (Tas.) | Tasmania | 844 | 14 | 0 | 62 | 1.6% | 0.0% | 7.4% |
| Cuballing | Western Australia | 932 | 15 | 0 | 68 | 1.6% | 0.0% | 7.2% |
| West Tamar | Tasmania | 23,300 | 374 | 6 | 1,682 | 1.6% | 0.0% | 7.2% |
| Northern Midlands | Tasmania | 14,223 | 227 | 9 | 940 | 1.6% | 0.1% | 6.6% |
| West Arthur | Western Australia | 805 | 13 | 0 | 56 | 1.6% | 0.0% | 6.9% |
| Dorset | Tasmania | 6,615 | 104 | 2 | 489 | 1.6% | 0.0% | 7.4% |
| Maranoa | Queensland | 13,107 | 206 | 8 | 871 | 1.6% | 0.1% | 6.6% |
| Blayney | New South Wales | 7,581 | 118 | 5 | 540 | 1.6% | 0.1% | 7.1% |
| Chapman Valley | Western Australia | 1,801 | 28 | 1 | 118 | 1.5% | 0.0% | 6.5% |
| Southern Mallee | South Australia | 2,014 | 31 | 1 | 152 | 1.5% | 0.1% | 7.5% |
| Broomehill-Tambellup | Western Australia | 1,091 | 16 | 0 | 73 | 1.5% | 0.0% | 6.7% |
| Orroroo Carrieton | South Australia | 912 | 13 | 0 | 55 | 1.4% | 0.0% | 6.0% |
| Break O'Day | Tasmania | 6,436 | 93 | 1 | 422 | 1.4% | 0.0% | 6.6% |
| Boyup Brook | Western Australia | 1,938 | 28 | 1 | 123 | 1.4% | 0.0% | 6.4% |
| Three Springs | Western Australia | 586 | 8 | 0 | 36 | 1.4% | 0.0% | 6.1% |
| Goyder | South Australia | 4,142 | 59 | 1 | 290 | 1.4% | 0.0% | 7.0% |
| Bogan | New South Wales | 2,462 | 35 | 1 | 165 | 1.4% | 0.0% | 6.7% |
| Cunderdin | Western Australia | 1,346 | 19 | 0 | 80 | 1.4% | 0.0% | 6.0% |
| Mount Remarkable | South Australia | 2,819 | 38 | 1 | 170 | 1.4% | 0.0% | 6.0% |
| Latrobe (Tas.) | Tasmania | 12,184 | 163 | 2 | 749 | 1.3% | 0.0% | 6.1% |
| Charters Towers | Queensland | 12,017 | 158 | 7 | 620 | 1.3% | 0.1% | 5.2% |
| Central Highlands (Tas.) | Tasmania | 2,515 | 33 | 2 | 136 | 1.3% | 0.1% | 5.4% |
| Oberon | New South Wales | 5,543 | 73 | 3 | 352 | 1.3% | 0.1% | 6.4% |
| Bridgetown-Greenbushes | Western Australia | 5,524 | 71 | 2 | 329 | 1.3% | 0.0% | 6.0% |
| Armidale | New South Wales | 29,392 | 378 | 18 | 1,595 | 1.3% | 0.1% | 5.4% |
| Bulloo | Queensland | 341 | 4 | 0 | 18 | 1.3% | 0.0% | 5.2% |
| Walcha | New South Wales | 2,954 | 37 | 2 | 161 | 1.3% | 0.1% | 5.4% |
| Glenorchy | Tasmania | 48,426 | 603 | 5 | 2,764 | 1.2% | 0.0% | 5.7% |
| Carnarvon | Western Australia | 5,145 | 64 | 5 | 196 | 1.2% | 0.1% | 3.8% |
| Charles Sturt | South Australia | 131,690 | 1,628 | 25 | 7,195 | 1.2% | 0.0% | 5.5% |
| Peppermint Grove | Western Australia | 1,724 | 21 | 0 | 87 | 1.2% | 0.0% | 5.1% |
| Brookton | Western Australia | 954 | 12 | 0 | 52 | 1.2% | 0.0% | 5.4% |
| Circular Head | Tasmania | 8,158 | 98 | 1 | 445 | 1.2% | 0.0% | 5.5% |
| Uralla | New South Wales | 5,910 | 71 | 3 | 331 | 1.2% | 0.1% | 5.6% |
| Broken Hill | New South Wales | 17,569 | 207 | 5 | 858 | 1.2% | 0.0% | 4.9% |
| Cleve | South Australia | 1,753 | 20 | 0 | 87 | 1.1% | 0.0% | 5.0% |
| Mapoon | Queensland | 236 | 3 | 0 | 12 | 1.1% | 0.1% | 5.1% |
| Meander Valley | Tasmania | 20,313 | 229 | 9 | 1,031 | 1.1% | 0.0% | 5.1% |
| Boddington | Western Australia | 1,755 | 19 | 0 | 87 | 1.1% | 0.0% | 4.9% |
| Unincorporated Vic | Victoria | 947 | 10 | 0 | 48 | 1.1% | 0.0% | 5.0% |
| Karratha | Western Australia | 23,771 | 255 | 15 | 850 | 1.1% | 0.1% | 3.6% |
| Woodanilling | Western Australia | 471 | 5 | 0 | 22 | 1.1% | 0.0% | 4.7% |
| Kojonup | Western Australia | 1,970 | 21 | 0 | 93 | 1.1% | 0.0% | 4.7% |
| Croydon | Queensland | 269 | 3 | 0 | 10 | 1.0% | 0.1% | 3.8% |
| Derwent Valley | Tasmania | 11,378 | 117 | 3 | 524 | 1.0% | 0.0% | 4.6% |
| Beverley | Western Australia | 1,736 | 18 | 0 | 80 | 1.0% | 0.0% | 4.6% |
| Etheridge | Queensland | 724 | 7 | 0 | 28 | 1.0% | 0.0% | 3.9% |
| Flinders (Qld) | Queensland | 1,539 | 16 | 0 | 63 | 1.0% | 0.0% | 4.1% |
| Carnamah | Western Australia | 563 | 6 | 0 | 25 | 1.0% | 0.0% | 4.4% |
| Roper Gulf | Northern Territory | 7,488 | 73 | 4 | 297 | 1.0% | 0.0% | 4.0% |
| Central Darling | New South Wales | 1,744 | 17 | 1 | 63 | 1.0% | 0.0% | 3.6% |
| Clarence | Tasmania | 56,624 | 527 | 4 | 2,425 | 0.9% | 0.0% | 4.3% |
| Brighton | Tasmania | 16,951 | 151 | 2 | 701 | 0.9% | 0.0% | 4.1% |
| Jerramungup | Western Australia | 1,190 | 10 | 0 | 45 | 0.9% | 0.0% | 3.8% |
| Queenscliffe | Victoria | 2,572 | 22 | 0 | 91 | 0.8% | 0.0% | 3.5% |
| Hobart | Tasmania | 55,550 | 456 | 4 | 2,112 | 0.8% | 0.0% | 3.8% |
| Glen Innes Severn | New South Wales | 8,942 | 72 | 3 | 366 | 0.8% | 0.0% | 4.1% |
| Murweh | Queensland | 3,972 | 32 | 1 | 144 | 0.8% | 0.0% | 3.6% |
| Pingelly | Western Australia | 1,070 | 9 | 0 | 38 | 0.8% | 0.0% | 3.6% |
| Wandering | Western Australia | 545 | 4 | 0 | 20 | 0.8% | 0.0% | 3.7% |
| Narembeen | Western Australia | 821 | 6 | 0 | 28 | 0.8% | 0.0% | 3.4% |
| Goomalling | Western Australia | 980 | 8 | 0 | 34 | 0.8% | 0.0% | 3.5% |
| Wudinna | South Australia | 1,178 | 9 | 0 | 37 | 0.8% | 0.0% | 3.2% |
| Waratah-Wynyard | Tasmania | 13,074 | 100 | 1 | 452 | 0.8% | 0.0% | 3.5% |
| Gnowangerup | Western Australia | 1,258 | 10 | 0 | 41 | 0.8% | 0.0% | 3.2% |
| Doomadgee | Queensland | 1,425 | 11 | 0 | 41 | 0.7% | 0.0% | 2.9% |
| Kent | Western Australia | 505 | 4 | 0 | 15 | 0.7% | 0.0% | 3.0% |
| Williams | Western Australia | 1,063 | 8 | 0 | 36 | 0.7% | 0.0% | 3.4% |
| Kingborough | Tasmania | 38,402 | 285 | 3 | 1,318 | 0.7% | 0.0% | 3.4% |
| Lake Grace | Western Australia | 1,289 | 9 | 0 | 37 | 0.7% | 0.0% | 2.9% |
| Coorow | Western Australia | 782 | 5 | 0 | 23 | 0.7% | 0.0% | 3.0% |
| Northampton | Western Australia | 3,338 | 23 | 1 | 95 | 0.7% | 0.0% | 2.8% |
| Shark Bay | Western Australia | 1,065 | 7 | 0 | 23 | 0.7% | 0.0% | 2.2% |
| Dowerin | Western Australia | 732 | 5 | 0 | 21 | 0.7% | 0.0% | 2.9% |
| Port Hedland | Western Australia | 15,995 | 106 | 5 | 388 | 0.7% | 0.0% | 2.4% |
| Winton | Queensland | 1,133 | 7 | 0 | 31 | 0.7% | 0.0% | 2.7% |
| Trayning | Western Australia | 307 | 2 | 0 | 8 | 0.6% | 0.0% | 2.7% |
| Narrogin | Western Australia | 4,949 | 31 | 1 | 148 | 0.6% | 0.0% | 3.0% |
| Tammin | Western Australia | 400 | 2 | 0 | 10 | 0.6% | 0.0% | 2.5% |
| West Coast | Tasmania | 4,050 | 24 | 1 | 92 | 0.6% | 0.0% | 2.3% |
| Barkly | Northern Territory | 7,160 | 42 | 1 | 162 | 0.6% | 0.0% | 2.3% |
| Robe | South Australia | 1,569 | 9 | 0 | 41 | 0.6% | 0.0% | 2.6% |
| Streaky Bay | South Australia | 2,139 | 12 | 0 | 55 | 0.6% | 0.0% | 2.6% |
| Glamorgan-Spring Bay | Tasmania | 4,057 | 24 | 0 | 111 | 0.6% | 0.0% | 2.7% |
| Coolgardie | Western Australia | 3,580 | 21 | 1 | 77 | 0.6% | 0.0% | 2.1% |
| Whyalla | South Australia | 21,430 | 124 | 3 | 535 | 0.6% | 0.0% | 2.5% |
| Kellerberrin | Western Australia | 1,174 | 7 | 0 | 28 | 0.6% | 0.0% | 2.4% |
| Mingenew | Western Australia | 424 | 2 | 0 | 10 | 0.6% | 0.0% | 2.4% |
| Flinders Ranges | South Australia | 1,678 | 9 | 0 | 39 | 0.6% | 0.0% | 2.3% |
| Unincorporated SA | South Australia | 3,701 | 20 | 1 | 89 | 0.6% | 0.0% | 2.4% |
| Sorell | Tasmania | 15,684 | 83 | 1 | 386 | 0.5% | 0.0% | 2.5% |
| Karoonda East Murray | South Australia | 995 | 5 | 0 | 30 | 0.5% | 0.0% | 3.0% |
| Richmond | Queensland | 778 | 4 | 0 | 15 | 0.5% | 0.0% | 1.9% |
| Bruce Rock | Western Australia | 1,019 | 5 | 0 | 21 | 0.5% | 0.0% | 2.1% |
| Barcoo | Queensland | 318 | 2 | 0 | 7 | 0.5% | 0.0% | 2.1% |
| Yilgarn | Western Australia | 1,196 | 6 | 0 | 23 | 0.5% | 0.0% | 1.9% |
| Dundas | Western Australia | 697 | 3 | 0 | 14 | 0.5% | 0.0% | 2.0% |
| Quairading | Western Australia | 985 | 4 | 0 | 20 | 0.5% | 0.0% | 2.0% |
| Kentish | Tasmania | 6,862 | 31 | 1 | 139 | 0.5% | 0.0% | 2.0% |
| Kalgoorlie-Boulder | Western Australia | 30,759 | 134 | 5 | 532 | 0.4% | 0.0% | 1.7% |
| Corrigin | Western Australia | 1,021 | 4 | 0 | 19 | 0.4% | 0.0% | 1.9% |
| Quilpie | Queensland | 705 | 3 | 0 | 12 | 0.4% | 0.0% | 1.8% |
| Ashburton | Western Australia | 7,780 | 33 | 1 | 122 | 0.4% | 0.0% | 1.6% |
| Elliston | South Australia | 892 | 4 | 0 | 17 | 0.4% | 0.0% | 1.9% |
| Murchison | Western Australia | 108 | 0 | 0 | 1 | 0.4% | 0.0% | 1.3% |
| Huon Valley | Tasmania | 17,731 | 75 | 2 | 341 | 0.4% | 0.0% | 1.9% |
| Dumbleyung | Western Australia | 693 | 3 | 0 | 12 | 0.4% | 0.0% | 1.8% |
| Wyalkatchem | Western Australia | 487 | 2 | 0 | 9 | 0.4% | 0.0% | 1.8% |
| Kondinin | Western Australia | 866 | 3 | 0 | 15 | 0.4% | 0.0% | 1.7% |
| Tasman | Tasmania | 2,236 | 8 | 0 | 39 | 0.4% | 0.0% | 1.8% |
| Wickepin | Western Australia | 707 | 3 | 0 | 12 | 0.4% | 0.0% | 1.6% |
| George Town | Tasmania | 6,716 | 25 | 0 | 113 | 0.4% | 0.0% | 1.7% |
| Southern Midlands | Tasmania | 6,926 | 25 | 1 | 122 | 0.4% | 0.0% | 1.8% |
| Napranum | Queensland | 609 | 2 | 0 | 9 | 0.3% | 0.0% | 1.4% |
| Kimba | South Australia | 1,039 | 3 | 0 | 15 | 0.3% | 0.0% | 1.4% |
| Central Coast (Tas.) | Tasmania | 21,149 | 67 | 1 | 333 | 0.3% | 0.0% | 1.6% |
| Koorda | Western Australia | 373 | 1 | 0 | 4 | 0.3% | 0.0% | 1.2% |
| Nungarin | Western Australia | 257 | 1 | 0 | 3 | 0.3% | 0.0% | 1.2% |
| Dalwallinu | Western Australia | 1,430 | 4 | 0 | 16 | 0.3% | 0.0% | 1.1% |
| Mount Marshall | Western Australia | 463 | 1 | 0 | 5 | 0.2% | 0.0% | 1.0% |
| Central Desert | Northern Territory | 4,173 | 10 | 0 | 40 | 0.2% | 0.0% | 1.0% |
| Halls Creek | Western Australia | 4,105 | 10 | 0 | 37 | 0.2% | 0.0% | 0.9% |
| Kulin | Western Australia | 781 | 2 | 0 | 8 | 0.2% | 0.0% | 1.0% |
| Merredin | Western Australia | 3,228 | 7 | 0 | 31 | 0.2% | 0.0% | 0.9% |
| Roxby Downs | South Australia | 4,105 | 9 | 0 | 32 | 0.2% | 0.0% | 0.8% |
| Perenjori | Western Australia | 652 | 1 | 0 | 5 | 0.2% | 0.0% | 0.8% |
| Exmouth | Western Australia | 3,291 | 7 | 0 | 21 | 0.2% | 0.0% | 0.6% |
| Bunbury | Western Australia | 33,321 | 65 | 2 | 309 | 0.2% | 0.0% | 0.9% |
| Peterborough | South Australia | 1,669 | 3 | 0 | 13 | 0.2% | 0.0% | 0.8% |
| Cue | Western Australia | 230 | 0 | 0 | 1 | 0.2% | 0.0% | 0.6% |
| Anangu Pitjantjatjara Yankunytjatjara | South Australia | 2,595 | 4 | 0 | 18 | 0.2% | 0.0% | 0.7% |
| Westonia | Western Australia | 252 | 0 | 0 | 2 | 0.2% | 0.0% | 0.7% |
| McKinlay | Queensland | 848 | 1 | 0 | 5 | 0.2% | 0.0% | 0.6% |
| Alice Springs | Northern Territory | 28,932 | 44 | 0 | 202 | 0.2% | 0.0% | 0.7% |
| East Pilbara | Western Australia | 10,308 | 15 | 1 | 56 | 0.1% | 0.0% | 0.5% |
| Mukinbudin | Western Australia | 591 | 1 | 0 | 3 | 0.1% | 0.0% | 0.5% |
| Burnie | Tasmania | 20,083 | 25 | 0 | 112 | 0.1% | 0.0% | 0.6% |
| Morawa | Western Australia | 668 | 1 | 0 | 3 | 0.1% | 0.0% | 0.5% |
| Ceduna | South Australia | 3,274 | 4 | 0 | 17 | 0.1% | 0.0% | 0.5% |
| Aurukun | Queensland | 1,131 | 1 | 0 | 6 | 0.1% | 0.0% | 0.5% |
| Yalgoo | Western Australia | 365 | 0 | 0 | 1 | 0.1% | 0.0% | 0.3% |
| Unincorporated NSW | New South Wales | 988 | 1 | 0 | 4 | 0.1% | 0.0% | 0.4% |
| MacDonnell | Northern Territory | 6,624 | 5 | 0 | 23 | 0.1% | 0.0% | 0.3% |
| Pormpuraaw | Queensland | 633 | 0 | 0 | 2 | 0.1% | 0.0% | 0.3% |
| Devonport | Tasmania | 24,854 | 18 | 0 | 85 | 0.1% | 0.0% | 0.3% |
| Leonora | Western Australia | 1,720 | 1 | 0 | 4 | 0.1% | 0.0% | 0.3% |
| Boulia | Queensland | 477 | 0 | 0 | 1 | 0.1% | 0.0% | 0.2% |
| Mount Magnet | Western Australia | 701 | 0 | 0 | 1 | 0.1% | 0.0% | 0.2% |
| Sandstone | Western Australia | 117 | 0 | 0 | 0 | 0.1% | 0.0% | 0.2% |
| Upper Gascoyne | Western Australia | 187 | 0 | 0 | 0 | 0.0% | 0.0% | 0.1% |
| Laverton | Western Australia | 1,433 | 0 | 0 | 2 | 0.0% | 0.0% | 0.1% |
| Diamantina | Queensland | 268 | 0 | 0 | 0 | 0.0% | 0.0% | 0.1% |
| Wiluna | Western Australia | 572 | 0 | 0 | 1 | 0.0% | 0.0% | 0.1% |
| Ngaanyatjarraku | Western Australia | 1,478 | 0 | 0 | 1 | 0.0% | 0.0% | 0.1% |
| Torres | Queensland | 2,839 | 1 | 0 | 2 | 0.0% | 0.0% | 0.1% |
| Meekatharra | Western Australia | 1,293 | 0 | 0 | 1 | 0.0% | 0.0% | 0.1% |
| Menzies | Western Australia | 570 | 0 | 0 | 0 | 0.0% | 0.0% | 0.1% |
| Coober Pedy | South Australia | 1,572 | 0 | 0 | 1 | 0.0% | 0.0% | 0.0% |
| Maralinga Tjarutja | South Australia | 134 | 0 | 0 | 0 | 0.0% | 0.0% | 0.0% |
