# Supplementary material for: Ecological suitability of Japanese encephalitis virus in Australia: A modelling analysis of vector-host transmission dynamics to potential spillover in humans
Source: PLoS Negl Trop Dis. 2025 Nov 17;19(11):e0013722. doi: 10.1371/journal.pntd.0013722 (PMC12654935; doi:10.1371/journal.pntd.0013722)
Supplement: Attachment — Submitted filename: renamed_cd78b.pdf [file pntd.0013722.s009.pdf]

Review of “Ecological suitability of Japanese encephalitis virus in Australia: a modelling analysis of vector-host transmission dynamics to potential spillover in humans” with Manuscript ID PNTD-D-25-00710

Summary Assessment

This scientifically rigorous and well-structured manuscript by Skinner & Sartorius et al. presents a comprehensive spatial modelling analysis of Japanese encephalitis virus (JEV) transmission potential across Australia, integrating ecological, entomological, and human demographic data. By combining host and vector presence, vector competence, and human population density into a spatial framework, the authors calculate both an ecological suitability index and a spillover risk estimate. The study is timely, methodologically sound, and provides valuable insights for public health preparedness and surveillance.

The modelling approach is well-founded and benefits from empirical data sources and established frameworks. The clarity of writing is generally strong, though minor grammatical and stylistic refinements are suggested throughout the manuscript. The work offers a meaningful advancement in understanding JEV ecology and human risk within Australia and should be of interest to researchers, epidemiologists, and policymakers alike.

General Notes

The manuscript addresses a timely and important topic concerning the potential establishment and spread of JEV in Australia. The modelling approach is robust and integrates ecological, epidemiological, and demographic data in a clear and structured manner. The manuscript is generally well written, though several grammatical, syntactic, and stylistic corrections are recommended to improve clarity and precision. Below is a detailed, line-by-line review with scientific, grammatical, and logical feedback.

Abstract

line 16 "Japanese Encephalitis virus" should be: "Japanese encephalitis virus" (lowercase).

line 17 Rephrase for clarity: "...between *Culex* mosquitoes and waterbirds, with occasional spillover to humans."

line 18–20 I suggest rewording: "...confined to tropical northern Australia until 2021 after which outbreaks occurred for the first time in temperate southern regions in 2022 and 2023 respectively."

line 21 I suggest replacing "now endemic" with "potentially becoming endemic."

line 22-23 deleoped and applied

line 25–27 Avoid repetition of "developed a modelling framework." Combine with previous sentence.

line 33 Typo: "total population at that could be exposed" → "total population that could be exposed."

line 33–35 I suggest to introduce a abbreviation for readability: "...compared Local Government Areas (LGAs) with confirmed clinical cases to those without." This should then be incooperated troughout. LGA is not introduced as a abbreviation and troughout the fisr part of the manuscript “Local Government Areas” is used, whereas in the later part it is switched to LGAs. Please make sure the use is consistant. After the first introduction of the abbreviation, only the abbreviation should be used.

line 38–39 Potentially split for readability: "If JEV were to become established in ecologically suitable areas, high spillover potential would exist along Australia's eastern coast."

line 45 Consider replacing "model’s ability" with "model’s capability."

Introduction

line 58–60 Consider "central nervous system infection" instead of "nervous system infection."

line 66–68 Reword slightly: "...making the prediction of environmental and climatic spillover conditions critical to guiding interventions."

line 69 Consider replacing "becoming an escalating" with "emerging."

line 70 Clarify to "Prior to its designation as a nationally notifiable disease."

line 83 Consider rephrasing as: "...suggesting that endemic transmission may already be established."

line 86 Consider: "...suggesting potential for urban establishment."

Results

line 132–145 Consider replacing "elevated probability" with "increased probability".

line 206–211 Consider clarifying how "population-weighted mean" was calculated.

Discussion

line 219–224 Accurate summary of advancement over previous studies.

line 226–227 I suggest clarifying what is meant by: "...each process is necessary but not sufficient to drive outbreaks alone."

line 231–233 Emphasize that some areas of past outbreaks were not captured, and discuss potential data limitations.

line 235–239 Excellent discussion of model uncertainty. Suggest adding a sentence on how future data could resolve this.

line 244–246 Reiterate that model outputs identify potential, not actual, transmission.

line 255 Typo: "people at have the potential" → "people at risk of acidental spillover,"

line 259–260 "broadens the areas considered at suitable" → "broadens the areas considered suitable."

line 268–270 Good point regarding piggery-associated interventions. Clarify if this is assumed or supported by data.

line 277–284 Clear differentiation of model uses. Could benefit from example use cases (e.g., resource allocation support etc.).

line 289–299 Important limitations stated transparently. Suggest briefly referencing possible seasonal modeling approaches.

line 307–309 Valuable note on future species-level modeling.

line 314–316 Strong conclusion on utility of mechanistic models despite data limitations.

Conclusions

line 318–319 Good framing of recent outbreaks and the knowledge gap.

line 321–324 Clear and actionable guidance. I suggest changing "presents an expanding threat" to "represents a growing threat."

line 329–332 Reiterate the need for integration of temporal and climate data.

Methods

line 335–347 The conceptual model is logically structured. Clarify whether vaccination data were available.

line 351–358 Strong justification of vector and host selection. Clearly state rationale for excluding bats and ducks.

line 362–370 Suggest expanding on the independence assumption between vector-host combinations.

line 371–375 Good aggregation approach.

line 383–397 Use of bloodmeal data is appropriate. Consider noting possible biases in geographic sampling.

line 400–405 Well executed niche modeling description. Clarify NDVI time range.

line 417–426 Clear description of human population modeling.

line 428–430 Mapping tools are appropriate.

line 432–442 Sensible validation method given sparse outbreak data.
