# Supplementary material for: Ecological suitability of Japanese encephalitis virus in Australia: A modelling analysis of vector-host transmission dynamics to potential spillover in humans
Source: PLoS Negl Trop Dis. 2025 Nov 17;19(11):e0013722. doi: 10.1371/journal.pntd.0013722 (PMC12654935; doi:10.1371/journal.pntd.0013722)
Supplement: Attachment — Submitted filename: renamed_ff665.pdf [file pntd.0013722.s011.pdf]

Review of "Ecological suitability of Japanese encephalitis virus in Australia: a modelling analysis of vector-host transmission dynamics to potential spillover in humans" with Manuscript ID PNTD-D-25-00710_R1

The authors have thoroughly revised the manuscript and addressed virtually all prior reviewer comments with precision. The revisions strengthen methodological clarity, conceptual framing, and policy relevance. Most stylistic, structural, and scientific issues from my first review have been implemented. The writing is clearer, the modelling description is expanded, and methodological assumptions and data availability are now precise. Only minor clarifications remain regarding numerical model validation, summarization of key variable definitions, and optional graphical representation of uncertainty. Subject to these small refinements, the revised manuscript is suitable for acceptance.

Abstract

- The capitalization, sentence clarity, and abbreviation consistency (LGA) corrections previously flagged have all been implemented correctly.
- The authors integrated the "potentially becoming endemic" phrasing and reorganized the sentences for readability.
- All abstract-related points were fully addressed.

Introduction

- All linguistic edits and conceptual suggestions (e.g., "central nervous system infection," clarification of national notification status, urban establishment note) have been executed.
- Updated references now include 2024–2025 outbreak and surveillance data, demonstrating that the authors also refreshed the epidemiological context.
- Fully addressed; section now stronger and updated.

Methods

- The Methods section has undergone extensive expansion.
  - Vaccination data explanation is now explicitly included.
  - Exclusion rationale for bats and ducks is clearly written.
  - The independence assumption between vector–host combinations is formally described mathematically (Vunion formula and statistical assumption).
  - NDVI temporal range (October 2018–March 2019) was inserted.
  - Bloodmeal data source and bias discussion were added.
- Only minor readability adjustments might still be beneficial (e.g., summarizing definitions of PP, BP, IP, TP in a short table for the reader).
- Fully addressed, with optional stylistic enhancement noted.

Results

- The updated Results now properly define how the population-weighted mean is calculated, as requested.

- Table 1 provides national and sub-national spillover estimates with explicit 95% confidence intervals; this clarifies earlier ambiguity.
- Mapping outputs (Figures 2–5) directly visualize recommendations you raised.
- All analytics and interpretation feedback were implemented.

Discussion

- The key conceptual clarification ("each process necessary but not sufficient") was rewritten precisely as suggested.
- Discussion now explicitly recognizes that not all historic outbreak areas were captured due to ecological and data limitations.
- Additional explanation is inserted specifying that model outputs indicate potential, not actual transmission.
- They also included your recommendation for future data and seasonal modeling.
- Minor further improvement could be in emphasizing model validation metrics (e.g., AUC or other numerical indicators) — current text lists $P < 0.001$ comparison, but lacks summary statistic.
- Fully addressed conceptually; small numeric validation detail could be added for completeness.

Conclusions

- Wording revisions ("represents a growing threat") and integration of climate/temporal data for future iterations were implemented.
- The conclusions now directly mirror actionable outputs and integration of empirical data.
- Fully addressed.

Minor and Editorial Aspects

- Typographic corrections and all grammatical cleanups flagged are implemented.
- The authors also improved figure captions and licensing notes in response to editorial requirements, ensuring compliance with CC-BY sources.
- Clarity and structure markedly improved; only minor tightening of long sentences could enhance readability in the Discussion, but not absolutely necessary.

Follow-up Suggestions

1. **Quantitative Validation Reporting:**
   Include at least one numeric performance metric (AUC, R^2, or sensitivity/specificity) to complement the "statistically higher LGA mean value" test for clearer validation strength.
2. **Definition Summary Table:**
   A compact table summarizing model variables (PP, BP, IP, TP, Vunion, etc.) would improve clarity, especially for interdisciplinary readers.
3. **Uncertainty Quantification:**
   The Discussion references uncertainty intervals but does not illustrate them in main

figures. Suggest adding shaded confidence bounds or explicit mean ± 95 % CI in Supplementary Figures.

4. **Contextual Integration:**
   While updates to surveillance and 2025 events are excellent, the authors could add one bridging paragraph connecting this ecological model to current national surveillance and vaccination policies for enhanced applied relevance.
