# Supplementary material for: Ecological suitability of Japanese encephalitis virus in Australia: A modelling analysis of vector-host transmission dynamics to potential spillover in humans
Source: PLoS Negl Trop Dis. 2025 Nov 17;19(11):e0013722. doi: 10.1371/journal.pntd.0013722 (PMC12654935; doi:10.1371/journal.pntd.0013722)
Supplement: Attachment — Submitted filename: 120825_response_to_reviewers.docx [file pntd.0013722.s010.docx]

* A rebuttal letter that responds to each point raised by the editor and reviewer(s). You should upload this letter as a separate file labeled 'Response to Reviewers'. This file does not need to include responses to any formatting updates and technical items listed in the 'Journal Requirements' section below.
* A marked-up copy of your manuscript that highlights changes made to the original version. You should upload this as a separate file labeled 'Revised Manuscript with Track Changes'.
* An unmarked version of your revised paper without tracked changes. You should upload this as a separate file labeled 'Manuscript'.

**Additional Editor Comments (if provided):**

Please revise your manuscript, taking into consideration the reviewers' suggestions. Ensure that all comments are addressed thoroughly and that any changes are clearly indicated in your revised submission.

We would like to thank the reviewers for their comments. We have addressed each and respond to them individually below.

**Journal Requirements:**

1) We ask that a manuscript source file is provided at Revision. Please upload your manuscript file as a .doc, .docx, .rtf or .tex. If you are providing a .tex file, please upload it under the item type u2018LaTeX Source Fileu2019 and leave your .pdf version as the item type u2018Manuscriptu2019.

2) Please provide an Author Summary. This should appear in your manuscript between the Abstract (if applicable) and the Introduction, and should be 150-200 words long. The aim should be to make your findings accessible to a wide audience that includes both scientists and non-scientists. Sample summaries can be found on our website under Submission Guidelines:
<https://journals.plos.org/plosntds/s/submission-guidelines#loc-parts-of-a-submission>

Author summary has been added

3) Please upload all main figures as separate Figure files in .tif or .eps format. For more information about how to convert and format your figure files please see our guidelines: 
<https://journals.plos.org/plosntds/s/figures>

4) We notice that your supplementary Figures, and Tables are included in the manuscript file. Please remove them and upload them with the file type 'Supporting Information'. Please ensure that each Supporting Information file has a legend listed in the manuscript after the references list.

5) Some material included in your submission may be copyrighted. According to PLOSu2019s copyright policy, authors who use figures or other material (e.g., graphics, clipart, maps) from another author or copyright holder must demonstrate or obtain permission to publish this material under the Creative Commons Attribution 4.0 International (CC BY 4.0) License used by PLOS journals. Please closely review the details of PLOSu2019s copyright requirements here: PLOS Licenses and Copyright. If you need to request permissions from a copyright holder, you may use PLOS's Copyright Content Permission form.
Please respond directly to this email and provide any known details concerning your material's license terms and permissions required for reuse, even if you have not yet obtained copyright permissions or are unsure of your material's copyright compatibility. Once you have responded and addressed all other outstanding technical requirements, you may resubmit your manuscript within Editorial Manager. 
Potential Copyright Issues:
i) Figure 1 appears to have been previously published. Please provide written permission from the copyright holder to publish this under our CC-BY 4.0 license, or remove the figure / replace the image. Please note we do not recommend using standard request forms available on Publishers' websites, as they grant single use rather than republication under an open access license.

Thank you for your comment. Figure 1 is an original map created by the authors, adapted from McGuinness SL and Lau CL (2023), published in the *Journal of Travel Medicine* by Oxford University Press under a Creative Commons CC BY license. As this license permits unrestricted reuse with appropriate attribution, no additional permission is required. Substantial modifications were made in our adaptation, including updated time periods, revised symbology, and the inclusion of new data layers. We have updated the figure legend to include proper citation and attribution consistent with CC BY 4.0 requirements.

ii) Figures 1, 2, 3, 4, 5, and S3-S6. Please (a) provide a direct link to the base layer of the map (i.e., the country or region border shape) and ensure this is also included in the figure legend; and (b) provide a link to the terms of use / license information for the base layer image or shapefile. We cannot publish proprietary or copyrighted maps (e.g. Google Maps, Mapquest) and the terms of use for your map base layer must be compatible with our CC BY 4.0 license.
Note: if you created the map in a software program like R or ArcGIS, please locate and indicate the source of the basemap shapefile onto which data has been plotted.
If your map was obtained from a copyrighted source please amend the figure so that the base map used is from an openly available source. Alternatively, please provide explicit written permission from the copyright holder granting you the right to publish the material under our CC BY 4.0 license.
If you are unsure whether you can use a map or not, please do reach out and we will be able to help you. The following websites are good examples of where you can source open access or public domain maps:
* U.S. Geological Survey (USGS) - All maps are in the public domain. (<http://www.usgs.gov>)
* PlaniGlobe - All maps are published under a Creative Commons license so please cite u201cPlaniGlobe, <http://www.planiglobe.com>, CC BY 2.0u201d in the image credit after the caption. (<http://www.planiglobe.com/?lang=enl>)
* Natural Earth - All maps are public domain. (<http://www.naturalearthdata.com/about/terms-of-use/>).

All figure captions have been updated accordingly.

6) We note that your Data Availability Statement is currently as follows: "All relevant data used in this study are clearly referenced with source links to where these data can be accessed.". Please confirm at this time whether or not your submission contains all raw data required to replicate the results of your study. Authors must share the “minimal data set” for their submission. PLOS defines the minimal data set to consist of the data required to replicate all study findings reported in the article, as well as related metadata and methods (<https://journals.plos.org/plosone/s/data-availability#loc-minimal-data-set-definition>).
For example, authors should submit the following data: 
1) The values behind the means, standard deviations and other measures reported;
2) The values used to build graphs;
3) The points extracted from images for analysis..
Authors do not need to submit their entire data set if only a portion of the data was used in the reported study.
If your submission does not contain these data, please either upload them as Supporting Information files or deposit them to a stable, public repository and provide us with the relevant URLs, DOIs, or accession numbers. For a list of recommended repositories, please see <https://journals.plos.org/plosone/s/recommended-repositories>. 
If there are ethical or legal restrictions on sharing a de-identified data set, please explain them in detail (e.g., data contain potentially sensitive information, data are owned by a third-party organization, etc.) and who has imposed them (e.g., an ethics committee). Please also provide contact information for a data access committee, ethics committee, or other institutional body to which data requests may be sent. If data are owned by a third party, please indicate how others may request data access.

We have updated our data availability statement and included a publicly available github repository with code which generates all results.

“All data used in this study are openly available from the sources cited in the manuscript. All code used to process the data, construct the indices and generate the results is openly available at: <https://github.com/ebskinner/JEV-spillover-risk>.”

7) Please amend your detailed Financial Disclosure statement. This is published with the article. It must therefore be completed in full sentences and contain the exact wording you wish to be published.
1) State what role the funders took in the study. If the funders had no role in your study, please state: "The funders had no role in study design, data collection and analysis, decision to publish, or preparation of the manuscript."
2) If any authors received a salary from any of your funders, please state which authors and which funders..
If you did not receive any funding for this study, please simply state: u201cThe authors received no specific funding for this work.u201d

Updated with 1) as suggested.

8) Please send a completed 'Competing Interests' statement, including any COIs declared by your co-authors. If you have no competing interests to declare, please state "The authors have declared that no competing interests exist". Otherwise please declare all competing interests beginning with the statement "I have read the journal's policy and the authors of this manuscript have the following competing interests".

This has been updated with the following, as suggested:

“The authors have declared that no competing interests exist"

**Reviewers' Comments:**

Reviewer's Responses to Questions

**Key Review Criteria Required for Acceptance?**

As you describe the new analyses required for acceptance, please consider the following:

**Methods**

-Are the objectives of the study clearly articulated with a clear testable hypothesis stated?

-Is the study design appropriate to address the stated objectives?

-Is the population clearly described and appropriate for the hypothesis being tested?

-Is the sample size sufficient to ensure adequate power to address the hypothesis being tested?

-Were correct statistical analysis used to support conclusions?

-Are there concerns about ethical or regulatory requirements being met?

Reviewer #1: The objectives of the study are clearly articulated, focusing on the development of a spatially explicit modelling framework to estimate ecological suitability for Japanese encephalitis virus (JEV) transmission and potential human spillover across Australia. Although a formal hypothesis is not explicitly stated in a traditional sense, the goals are specific, testable, and well defined. The study design is appropriate for addressing these objectives, employing a mechanistic model that integrates ecological, entomological, and human demographic data in a spatial context. The populations under study—specifically the vectors (Cx. annulirostris and Cx. quinquefasciatus), hosts (Ardeidae birds, domestic and feral pigs), and human populations—are well described and relevant for the aims of the analysis.

The sample size, in terms of ecological and occurrence data points, is appropriate for the modelling approach and provides sufficient geographic and taxonomic coverage to ensure robustness. The statistical methods used are appropriate and well justified, including maximum entropy modelling, integration of vector-host contact and competence parameters, and population-weighted validation techniques. These methods adequately support the study's conclusions. No concerns were identified regarding ethical or regulatory compliance, as the study relies solely on publicly available and non-identifiable data, with a clear statement included regarding the role of funding sources and data transparency.

Thank you for your positive and thoughtful review. We appreciate your recognition of the clarity of our objectives, the appropriateness of our modelling approach, and the robustness of our data and methods. No changes were required in response to this comment.

Reviewer #2: - In the construction of any index, it is essential to explicitly state the possible range of values the index can assume, as well as the interpretation of those values. The authors should clarify these aspects for both the ecological suitability index and the human spillover potential index.

The ecological suitability index or combined vectorial potential probabilities to realise JEV transmission (V_union_) was calculated at a 1 km² resolution based on the combined vectorial potential *of Cx. annulirostris* and *Cx. quinquefasciatus*. Each component probability (PP, BP, IP, TP) ranged from 0 to 1, and multiplication implied an assumption of independence between these parameters, as empirical data on joint distributions are lacking. For each vector species, the total vectorial potential (Vi) could theoretically range from 0 to 1 (multiplicative process above would results in a combined probability that could theoretical range from 0 to 1 if perfect 0 and 1 probabilities for all input components), and hence the combined suitability index using set union (V_union_ = V_1_ + V_2_ − V_1_ × V_2_) could also only range theoretical range from 0 to just under 1. In ecological terms, values near 0 indicate minimal suitability for JEV transmission, reflecting low probability of vector or host presence, low contact rates, or low vector competence. Conversely, values approaching 1 represent the highest suitability, where competent vectors (*Cx. annulirostris* and *Cx. quinquefasciatus*) and amplifying hosts co-occur with maximum probability, contact, and transmission potential. However empiric data based on the literature for the contact rates, vector transmission competence etc as well as the occurrence probability modelling of the vectors and hosts are substantially lower than the theoretical maximum threshold of 100%. The range based on the empiric data varied from 0 to 0.18 i.e. the maximum vectorial potential for JEV transmission was 18%.

We have now included the following page 12 of the revised manuscript:

“All component probabilities used in calculating vectorial probability potential for JEV transmission (namely, the probability of vector presence (PP), bloodmeal preference (BP), infection probability (IP), and transmission probability (TP)) are theoretically bounded in probability space i.e. 0 and 1. These were combined multiplicatively for each host-vector interaction, under the simplifying assumption of independence between parameters due to the lack of empirical data on their joint distributions. For each mosquito species (Cx. annulirostris and Cx. quinquefasciatus), the total vectorial potential (Vi) was calculated as the sum of contributions from three host groups (Ardeidae birds, feral pigs, and domestic pigs). While the theoretical maximum for each *V_i_* could approach 1 under perfect conditions (i.e., all input probabilities equal to 1), the combined ecological suitability index (*V_union_*), calculated using standard probability union formula, also has a theoretical upper bound just below 1. In ecological terms, values near 0 indicate minimal suitability for JEV transmission, reflecting low probability of vector or host presence, low contact rates, or low vector competence. Values approaching 1 represent the highest suitability, where competent vectors and amplifying hosts co-occur with maximum probability, contact, and transmission potential. However, in practice, the empirically derived input parameters—based on literature estimates for contact rates, vector competence, and occurrence probabilities—are substantially lower than their theoretical maxima. As a result, the observed ecological suitability index values in our model ranged from 0 to 0.18, indicating that the maximum vectorial potential for JEV transmission under current ecological conditions is approximately 18%.”

Human spillover potential was calculated per 1 km² as the product of the ecological suitability index or combined vectorial potential for JEV transmission (*V_union_*) and human population density. Unlike *V_union_*, this index has no fixed upper bound, since values scale directly with local population size. Higher values of the spillover index indicate areas where high ecological suitability coincides with dense human populations, representing greater potential for human exposure should transmission occur. Importantly, even areas with moderate ecological suitability can yield high absolute population at risk if population density is sufficiently high. Lower values reflect either low ecological suitability, low population density, or both. Conversely, areas with high ecological suitability but low population density may have lower absolute spillover estimates.

- Figure 7 presents the structure used to construct the indices. It is recommended that the main text adopt the same notation as the equations and explicitly demonstrate how the probabilities shown in Figure 7 are derived. While the relevant information is provided in subsections, a direct connection with the formal notation is crucial for clarity and reproducibility.

Many thanks. Please see detailed response to this following on with linked comment below.

- In Figure 7, the formulas for each species (VA, VF, and VD) are calculated as the product of probabilities. This implies an assumption of independence between the events of transmission, infection, presence, and bloodfeeding. Is this assumption reasonable? If so, it should be explicitly stated in the text. Moreover, as these components are products of probabilities between 0 and 1, each individual term also ranges from 0 to 1. Consequently, the sum of VA, VF, and VD could range from 0 to 3. Why was equal weighting assigned to each of these components? Do the three features considered (Ardeidae birds, feral pigs, and domestic pigs) indeed contribute equally to vector potential? Additionally, the vector potentials calculated for Culex annulirostris and Culex quinquefasciatus are treated as independent. Is this assumption valid in the ecological context of Australia? If so, this should also be justified in the manuscript. If these interpretations are correct, then the ecological suitability index could range from –3 to 3. In that case, what do high values (either positive or negative) mean? And what about values close to zero? The same line of reasoning applies to the human spillover potential index. If these interpretations are not accurate, the authors should rigorously clarify the actual properties of the presented indicators.

We thank the reviewer for this detailed and constructive comment, which has helped us clarify both the link between our conceptual framework (Figure 7) and the main text, and the assumptions underlying our indices.

In the Methods section, we have now:

1. Adopted the exact notation from Figure 7 when describing the calculation of vectorial potential, ecological suitability index, and human spillover potential, where each probability term is defined and linked to the relevant sub-model and data source.
2. Explicitly stated the assumption of independence between the probabilities of presence, bloodfeeding, infection, and transmission. This assumption was necessary due to the absence of empirical data on joint distributions of these parameters.
3. Justified equal weighting of Ardeidae birds, feral pigs, and domestic pigs in the summation of vectorial potential. Empirical data to quantify the relative contribution of these hosts to JEV amplification in Australia are lacking, so equal weighting was adopted as a pragmatic approach that can be refined as new evidence emerges.
4. Justified the assumption of independence between vector species when calculating the ecological suitability index (*V_union=_V_1_+V_2_−V_1_×V_2_*). This is reasonable in the Australian ecological context because *Culex annulirostris* and *Culex quinquefasciatus* have distinct habitat preferences (freshwater vs peri-urban), and while there is some spatial overlap, their fine-scale distributions and host contact patterns differ.
5. Clarified the theoretical range and interpretation of the indices:
   - Each vector-host probability potential ranges from 0 to 1.
   - Each vector species probability potential can range from 0 to 1.
   - The fundamental properties of probability union formula constrain the ecological suitability probability (V_union_) to probability space i.e. 0 to 1.

- Low values indicate minimal potential for JEV transmission due to low co-occurrence or competence of vectors and hosts; high values indicate greater co-occurrence and competence.
- The human spillover potential index has no fixed upper bound because V_union_ is multiplied by human population density.

- The interpretation of intermediate values leading to the final indices should be discussed in conjunction with the results shown in Figures 3 and 4. For example, subfigures A) and B) of Figure 3 are not directly comparable, as their color scales have different maximum values: the dark red in A) does not represent the same intensity as in B). This difference should be standardized or justified to allow meaningful comparisons.

We have retained the species-specific scales because the vectorial potential for *Cx. annulirostris* and *Cx. quinquefasciatus* were calculated independently, with species-specific input parameters for infection probability, transmission probability, and blood feeding preference. Standardising the scale would visually obscure important differences in each species’ spatial pattern by compressing the variation in one species to match the other. We have added a sentence to the figure legend clarifying that the scales are species-specific and values are not intended for direct comparison of magnitude between panels.

- In the legend of Figure 4, the term “vector-host transmission risk” is used. However, the map actually displays the ecological suitability index. It is important to clarify that this index does not directly measure transmission risk, nor is a formal definition of “risk” provided in the manuscript. A similar issue occurs in Table 1. Furthermore, although the values shown on the maps range from 0 to 1, this is not necessarily guaranteed by the model described in Figure 7, and this consistency needs to be revised or justified.

The legend has been updated and as you note we are not examining JEV risk here, but rather ecological suitability for transmission. We have added a note in text that the index represents the relative suitability of ecological conditions for JEV transmission.

Please see response to your earlier comment in this regard. The ecological suitability index or combined vectorial potential probabilities to realise JEV transmission (V_union_) was calculated at a 1 km² resolution based on the combined vectorial potential *of Cx. annulirostris* and *Cx. quinquefasciatus*. Each component probability (PP, BP, IP, TP) ranged from 0 to 1, and multiplication implied an assumption of independence between these parameters, as empirical data on joint distributions are lacking. For each vector species, the total vectorial potential (Vi) could theoretically range from 0 to 1 (multiplicative process above would results in a combined probability that could theoretical range from 0 to 1 if perfect 0 and 1 probabilities for all input components), and hence the combined suitability index using set union (V_union_ = V_1_ + V_2_ − V_1_ × V_2_) could also only range theoretical range from 0 to just under 1. In ecological terms, values near 0 indicate minimal suitability for JEV transmission, reflecting low probability of vector or host presence, low contact rates, or low vector competence. Conversely, values approaching 1 represent the highest suitability, where competent vectors (*Cx. annulirostris* and *Cx. quinquefasciatus*) and amplifying hosts co-occur with maximum probability, contact, and transmission potential. However empiric data based on the literature for the contact rates, vector transmission competence etc as well as the occurrence probability modelling of the vectors and hosts are substantially lower than the theoretical maximum threshold of 100%. The range based on the empiric data varied from 0 to 0.18 i.e. the maximum vectorial potential for JEV transmission was 18%.

We have now included the following page 12 of the revised manuscript:

“All component probabilities used in calculating vectorial probability potential for JEV transmission (namely, the probability of vector presence (PP), bloodmeal preference (BP), infection probability (IP), and transmission probability (TP)) are theoretically bounded in probability space i.e. 0 and 1. These were combined multiplicatively for each host-vector interaction, under the simplifying assumption of independence between parameters due to the lack of empirical data on their joint distributions. For each mosquito species (Cx. annulirostris and Cx. quinquefasciatus), the total vectorial potential (Vi) was calculated as the sum of contributions from three host groups (Ardeidae birds, feral pigs, and domestic pigs). While the theoretical maximum for each *V_i_* could approach 1 under perfect conditions (i.e., all input probabilities equal to 1), the combined ecological suitability index (*V_union_*), calculated using standard probability union formula, also has a theoretical upper bound just below 1. In ecological terms, values near 0 indicate minimal suitability for JEV transmission, reflecting low probability of vector or host presence, low contact rates, or low vector competence. Values approaching 1 represent the highest suitability, where competent vectors and amplifying hosts co-occur with maximum probability, contact, and transmission potential. However, in practice, the empirically derived input parameters—based on literature estimates for contact rates, vector competence, and occurrence probabilities—are substantially lower than their theoretical maxima. As a result, the observed ecological suitability index values in our model ranged from 0 to 0.18, indicating that the maximum vectorial potential for JEV transmission under current ecological conditions is approximately 18%.”

Finally, the authors should state which software was used for data processing and index construction. It is strongly recommended that all data and code used in the analysis be made publicly available in a suitable repository.

We have explicitly listed all software used for data processing and index construction in the Methods (Mapping section). We already provide an open GitHub repository with all analysis code and have reiterated in the Data Availability Statement that all data sources are cited and publicly available.

**Results**

-Does the analysis presented match the analysis plan?

-Are the results clearly and completely presented?

-Are the figures (Tables, Images) of sufficient quality for clarity?

Reviewer #1: The results are clearly and comprehensively presented, and they align well with the stated modelling framework and analysis plan described in the Methods section. The construction and interpretation of the ecological suitability index and the human spillover potential are logically sequenced and directly derived from the described input data, sub-models, and computational procedures. The results offer clear insight into regional variation in risk, supported by quantitative estimates and confidence intervals at national and sub-national levels. Figures and tables are of high quality, with clear legends and sufficient resolution to support interpretation. Spatial maps of host and vector distributions, ecological suitability, and spillover potential are particularly helpful and visually effective. The inclusion of validation results—comparing population-weighted suitability values between affected and unaffected Local Government Areas—adds credibility to the model's predictive utility. Overall, the results are appropriately detailed and well structured, with figures and supplementary materials enhancing the clarity and strength of the findings.

We thank the reviewer for their positive assessment. No changes were made in response to this comment, as no revisions were required.

Reviewer #2: (No Response)

**Conclusions**

-Are the conclusions supported by the data presented?

-Are the limitations of analysis clearly described?

-Do the authors discuss how these data can be helpful to advance our understanding of the topic under study?

-Is public health relevance addressed?

Reviewer #1: The conclusions are well supported by the data presented and appropriately reflect the scope and findings of the analysis. The authors clearly describe the limitations of their modelling approach, including the static nature of the estimates, potential biases in ecological niche data, and uncertainties arising from limited empirical data on vector and host species distributions in Australia. These limitations are acknowledged transparently and constructively, with suggestions for future work to incorporate species-level models and temporal dynamics. The discussion effectively places the findings within the broader context of Japanese encephalitis virus emergence, offering clear insights into how this modelling framework advances understanding of ecological drivers and human spillover risk. Importantly, the public health relevance is strongly emphasized, particularly the value of identifying high-risk areas for targeted surveillance, vector control, and vaccination planning. The study provides a useful foundation for proactive interventions and risk mitigation in the face of ongoing environmental change.

We thank the reviewer for their positive assessment. No changes were made in response to this comment, as no revisions were required.

Reviewer #2: (No Response)

**Editorial and Data Presentation Modifications?**

Use this section for editorial suggestions as well as relatively minor modifications of existing data that would enhance clarity. If the only modifications needed are minor and/or editorial, you may wish to recommend “Minor Revision” or “Accept”.

Reviewer #1: Overall, there are relatively minor revisions that, once addressed, will strengthen the manuscript's presentation and accessibility without requiring substantive changes to its analyses or conclusions.

We thank the reviewer for their positive assessment. No changes were made in response to this comment, as no revisions were required.

Reviewer #2: (No Response)

**Summary and General Comments**

Use this section to provide overall comments, discuss strengths/weaknesses of the study, novelty, significance, general execution and scholarship. You may also include additional comments for the author, including concerns about dual publication, research ethics, or publication ethics. If requesting major revision, please articulate the new experiments that are needed.

Reviewer #1: This manuscript presents a timely and well-executed modelling analysis of Japanese encephalitis virus (JEV) transmission risk and potential human spillover across Australia. By integrating host and vector ecology, vector competence, environmental suitability, and human population distribution into a high-resolution spatial framework, the authors provide a valuable and novel tool for informing public health interventions. The modelling approach is methodologically sound, appropriately validated, and well aligned with the study's stated objectives. The manuscript is clearly written and demonstrates a strong command of ecological modelling and public health relevance. The work is particularly important in the context of recent JEV outbreaks in non-endemic areas of Australia, and it contributes significantly to understanding the drivers of virus emergence and potential persistence.

Strengths of the study include its mechanistic framework, the thoughtful selection of species and ecological inputs, and the clear connection between ecological suitability and real-world public health applications. The limitations are acknowledged transparently and do not detract from the overall value of the findings. There are no concerns regarding research or publication ethics, and all data sources appear appropriately cited and openly accessible. Minor editorial and structural improvements are recommended to improve clarity, but no new experiments or analyses are required. With these revisions, the manuscript will be suitable for publication and will make a strong contribution to the literature on vector-borne disease ecology and preparedness.

Thank you for the positive and supportive comments, we too think this will make a strong contribution to the literature on VBD ecology.

Reviewer #2: The article makes a valuable contribution to the understanding of vector-host transmission dynamics and the spillover potential of Japanese encephalitis virus (JEV) in Australia, drawing on methodologies previously established in the literature. The approach adopted incorporates key ecological processes involved in transmission between vectors and hosts, as well as the density and distribution of the human population, to estimate exposure potential. The manuscript is well written, and the results are clearly presented. However, certain conceptual characteristics and properties of the constructed indices need to be more clearly defined and discussed.

We thank the reviewer for their thoughtful comments on our manuscript and for acknowledging the clarity of the writing and presentation. In response to the request for clearer definition and discussion of the conceptual characteristics and properties of the constructed indices, we have revised the Methods to explicitly incorporate the notation from Figure 7, define all probability terms, and clarify the assumptions and value ranges for each index. We have also updated the Results to restate the meaning of low and high index values when first presenting the ecological suitability and spillover indices. These changes address the points raised while maintaining the structure and clarity of the manuscript.

PLOS authors have the option to publish the peer review history of their article ([what does this mean?](https://track.editorialmanager.com/CL0/https:%2F%2Fjournals.plos.org%2Fplosntds%2Fs%2Feditorial-and-peer-review-process%23loc-peer-review-history/1/010f019797f81e3c-c2a61501-90d4-4d09-933e-bf364e5b562d-000000/j39LjV7aWwsDkG4JzNPCZQ_AU4ZhEF1xONyJ8WOG6V8=216)). If published, this will include your full peer review and any attached files.

If you choose “no”, your identity will remain anonymous but your review may still be made public.

**Do you want your identity to be public for this peer review?** For information about this choice, including consent withdrawal, please see our [Privacy Policy](https://track.editorialmanager.com/CL0/https:%2F%2Fwww.plos.org%2Fprivacy-policy/1/010f019797f81e3c-c2a61501-90d4-4d09-933e-bf364e5b562d-000000/38uDdgZGgo3DgiyPInmRiUXR9ykx7Gto7_8W_saSiRk=216).

Reviewer #1: No

Reviewer #2: No

[NOTE: If reviewer comments were submitted as an attachment file, they will be attached to this email and accessible via the submission site. Please log into your account, locate the manuscript record, and check for the action link "View Attachments". If this link does not appear, there are no attachment files.]

**Figure resubmission:**

While revising your submission, please upload your figure files to the Preflight Analysis and Conversion Engine (PACE) digital diagnostic tool, <https://pacev2.apexcovantage.com/>. PACE helps ensure that figures meet PLOS requirements. To use PACE, you must first register as a user. Registration is free. Then, login and navigate to the UPLOAD tab, where you will find detailed instructions on how to use the tool. If you encounter any issues or have any questions when using PACE, please email PLOS at [figures@plos.org](mailto:figures@plos.org). Please note that Supporting Information files do not need this step. If there are other versions of figure files still present in your submission file inventory at resubmission, please replace them with the PACE-processed versions.

**Reproducibility:**

To enhance the reproducibility of your results, we recommend that authors of applicable studies deposit laboratory protocols in protocols.io, where a protocol can be assigned its own identifier (DOI) such that it can be cited independently in the future. Additionally, PLOS ONE offers an option to publish peer-reviewed clinical study protocols. Read more information on sharing protocols at <https://plos.org/protocols?utm_medium=editorial-email&utm_source=authorletters&utm_campaign=protocols>

**Summary Assessment**

This scientifically rigorous and well-structured manuscript by Skinner & Sartorius et al. presents a comprehensive spatial modelling analysis of Japanese encephalitis virus (JEV) transmission potential across Australia, integrating ecological, entomological, and human demographic data. By combining host and vector presence, vector competence, and human population density into a spatial framework, the authors calculate both an ecological suitability index and a spillover risk estimate. The study is timely, methodologically sound, and provides valuable insights for public health preparedness and surveillance.

The modelling approach is well-founded and benefits from empirical data sources and established frameworks. The clarity of writing is generally strong, though minor grammatical and stylistic refinements are suggested throughout the manuscript. The work offers a meaningful advancement in understanding JEV ecology and human risk within Australia and should be of interest to researchers, epidemiologists, and policymakers alike.

We thank the reviewers for their positive comments. We agree that this work provides valuable and timely insights by integrating ecological, entomological, and demographic data into a spatial framework for JEV risk.

**General Notes**

The manuscript addresses a timely and important topic concerning the potential establishment and spread of JEV in Australia. The modelling approach is robust and integrates ecological, epidemiological, and demographic data in a clear and structured manner. The manuscript is generally well written, though several grammatical, syntactic, and stylistic corrections are recommended to improve clarity and precision. Below is a detailed, line-by-line review with scientific, grammatical, and logical feedback.

We thank the reviewers for their thoughtful and detailed feedback. The suggested corrections have helped improve the clarity, precision, and overall readability of the manuscript.

**Abstract**

**line 16** "Japanese Encephalitis virus" should be: "Japanese encephalitis virus" (lowercase).

Updated as suggested

**line 17** Rephrase for clarity: "...between *Culex* mosquitoes and waterbirds, with occasional spillover to humans."

Updated as suggested

**line 18–20** I suggest rewording: "...confined to tropical northern Australia until 2021 after which outbreaks occurred for the first time in temperate southern regions in 2022 and 2023 respectively."

Updated as suggested

**line 21** I suggest replacing "now endemic" with "potentially becoming endemic."

Updated as suggested

**line 22-23** deleoped and applied

Updated as suggested

**line 25–27** Avoid repetition of "developed a modelling framework." Combine with previous sentence.

Updated as suggested

**line 33** Typo: "total population at that could be exposed" → "total population that could be exposed."

Updated as suggested

**line 33–35** I suggest to introduce a abbreviation for readability: "...compared Local Government Areas (LGAs) with confirmed clinical cases to those without." This should then be incooperated troughout. LGA is not introduced as a abbreviation and troughout the fisr part of the manuscript “Local Government Areas” is used, whereas in the later part it is switched to LGAs. Please make sure the use is consistant. After the first introduction of the abbreviation, only the abbreviation should be used.

Updated as suggested

**line 38–39** Potentially split for readability: "If JEV were to become established in ecologically suitable areas, high spillover potential would exist along Australia's eastern coast."

Updated as suggested

**line 45** Consider replacing "model’s ability" with "model’s capability."

Updated as suggested

**Introduction**

**line 58–60** Consider "central nervous system infection" instead of "nervous system infection."

Updated as suggested

**line 66–68** Reword slightly: "...making the prediction of environmental and climatic spillover conditions critical to guiding interventions."

Updated as suggested

**line 69** Consider replacing "becoming an escalating" with "emerging."

Updated as suggested

**line 70** Clarify to "Prior to its designation as a nationally notifiable disease."

Updated as suggested

**line 83** Consider rephrasing as: "...suggesting that endemic transmission may already be established."

Updated as suggested

**line 86** Consider: "...suggesting potential for urban establishment."

Updated as suggested

**Results**

**line 132–145** Consider replacing "elevated probability" with "increased probability".

Updated as suggested

**line 206–211** Consider clarifying how "population-weighted mean" was calculated.

Updated to include: “The population-weighted mean was calculated for each LGA by multiplying the ecological suitability index by human population density at the 1 km grid level, then averaging these values weighted by the population within the LGA.”

**Discussion**

**line 219–224** Accurate summary of advancement over previous studies.

Thank you for this acknowledgement.

**line 226–227** I suggest clarifying what is meant by: "...each process is necessary but not sufficient to drive outbreaks alone."

Updated as suggested to state: “These processes, such as the presence of competent vectors, suitable hosts and favourable environmental conditions, are each necessary components for transmission but cannot on their own sustain outbreaks. Only when these processes align can local transmission occur and lead to human spillover”

**line 231–233** Emphasize that some areas of past outbreaks were not captured, and discuss potential data limitations.

Updated to include: “This indicates that while the model can capture broad patterns of spillover potential, some areas of past outbreaks were not identified as highly suitable, likely reflecting limitations in the available ecological and demographic data or unmeasured local factors that may have influenced transmission.”

**line 235–239** Excellent discussion of model uncertainty. Suggest adding a sentence on how future data could resolve this.

The current text addresses this through: Distinguishing between these scenarios would require additional data, such as enhanced surveillance for undetected circulation, field assessments of vector and host abundance and exposure, and localised environmental data to better understand the conditions that might influence vector-host interactions and disease transmission.

**line 244–246** Reiterate that model outputs identify potential, not actual, transmission.

Updated as suggested to state: “We hypothesise that the regions that have high ecological suitability represent areas where JEV transmission could occur under favourable conditions (i.e. model outputs identify potential and not actual transmission) given the availability of interacting competent vectors and hosts.”

**line 255** Typo: "people at have the potential" → "people at risk of acidental spillover,"

Updated as suggested

**line 259–260** "broadens the areas considered at suitable" → "broadens the areas considered suitable."

Updated as suggested

**line 268–270** Good point regarding piggery-associated interventions. Clarify if this is assumed or supported by data.

Updated as suggested

**line 277–284** Clear differentiation of model uses. Could benefit from example use cases (e.g., resource allocation support etc.).

We thank the reviewer; however, specific operational case studies are not yet available due to the recent emergence of JEV in Australia, and the examples provided are intended as illustrative only.

**line 289–299** Important limitations stated transparently. Suggest briefly referencing possible seasonal modeling approaches.

Updated with: “Incorporating temporal scales in future studies (e.g. coupling the framework with seasonal climate forecasts, time-series analysis, or mechanistic models of vector population dynamics) would allow identification of seasonal hotspots, facilitating more dynamic assessments.”

**line 307–309** Valuable note on future species-level modeling.

Thank you for this acknowledgement.

**line 314–316** Strong conclusion on utility of mechanistic models despite data limitations.

Thank you for this acknowledgement.

**Conclusions**

**line 318–319** Good framing of recent outbreaks and the knowledge gap.

Thank you for this acknowledgement.

**line 321–324** Clear and actionable guidance. I suggest changing "presents an expanding threat" to "represents a growing threat."

Updated as suggested.

**line 329–332** Reiterate the need for integration of temporal and climate data.

Updated as follows: The ecological suitability index and human spillover potential developed in this study provide a foundation for future work and can be refined as additional empirical data become available. Improving the predictive power will require integrating temporal variability, climate and other environmental changes affecting reservoir host abundance and distribution, as well as shifts in human–animal contact patterns. Such integration would provide a more dynamic understanding of JEV transmission potential in Australia and support timely, targeted interventions.

**Methods**

**line 335–347** The conceptual model is logically structured. Clarify whether vaccination data were available.

Vaccination data were not available. This has been updated in the text as follows: Given the very low vaccine coverage in Australia, with programs limited to high-risk areas, and the lack of available data, vaccination was not included in the current estimates, and its limited uptake is unlikely to substantially affect transmission dynamics.

e.g. <https://www.health.nsw.gov.au/infectious/jev/pages/vaccination.aspx>; https://www.health.qld.gov.au/clinical-practice/guidelines-procedures/diseases-infection/immunisation/japanese-encephalitis-virus-vaccination-centres

**line 351–358** Strong justification of vector and host selection. Clearly state rationale for excluding bats and ducks.

Updated with: The potential contribution of other vertebrate hosts to JEV transmission was not considered due to current inconclusive evidence and lack of experimental infection data and virus detections in free-living populations. We have also added the following to the limitations section: While evidence is currently inconclusive, other vertebrate hosts could potentially contribute to JEV transmission in Australia and were thus not accounted for in our present estimates. As more evidence emerges, we will incorporate these data for these other potential vertebrate hosts in future iterations of this work.

**line 362–370** Suggest expanding on the independence assumption between vector-host combinations.

See comments to Reviewer 2 above, this has been updated as suggested.

**line 371–375** Good aggregation approach.

Thank you for this acknowledgement.

**line 383–397** Use of bloodmeal data is appropriate. Consider noting possible biases in geographic sampling.

We have added this to the limitation section as follows: Vector and host occurrence data, along with bloodmeal data —which form the basis of the probability of occurrence models used—are likely biased towards relatively more densely populated areas.

**line 400–405** Well executed niche modeling description. Clarify NDVI time range.

We used the latest available grid NDVI available from the Australian Bureau of Meteorology (BoM) which is averaged for the period 20181001-20190331. We have included this additional detail in the revised manuscript.

Source: http://www.bom.gov.au/climate/austmaps/about-ndvi-maps.shtml#:~:text=The%20Normalised%20Difference%20Vegetation%20Index,amount%20of%20live%20green%20vegetation.

**line 417–426** Clear description of human population modeling.

Thank you for this acknowledgement.

**line 428–430** Mapping tools are appropriate.

Thank you for this acknowledgement.

**line 432–442** Sensible validation method given sparse outbreak data.

Thank you for this acknowledgement.
